# When face-tracking meets social networks: a story of politics in news videos

**DOI:** 10.1007/s41109-016-0003-2

**Published:** 2016-06-01

**Authors:** Benjamin Renoust, Tetsuro Kobayashi, Thanh Duc Ngo, Duy-Dinh Le, Shin’Ichi Satoh

**Affiliations:** 1 National Institute of Informatics, 2-1-2 Hitotsubashi, Chiyoda-ku, Tokyo, 101-8430 Japan; 2 JFLI CNRS UMI 3527, 7-3-1, Hongo, Bunkyo-ku, The University of Tokyo, Tokyo, 103-0033 Japan; 3 City University of Hong Kong, 18 Tat Hong Avenue, Kowloon Tong, Hong Kong, RPC; 4 University of Information Technology, Vietnam National University, Quarter 6, Linh Trung Ward, Thu Duc District, Ho Chi Minh City, Vietnam

**Keywords:** News analysis, Social network analysis, Face detection and tracking, Multiplex, Dynamic, Politics, Japan

## Abstract

In the age of data processing, news videos are rich mines of information. After all, the news are essentially created to convey information to the public. But can we go beyond what is directly presented to us and see a wider picture? Many works already focus on what we can discover and understand from the analysis of years of news broadcasting. These analysis bring monitoring and understanding of the activity of public figures, political strategies, explanation and even prediction of critical media events. Such tools can help public figures in managing their public image, as well as support the work of journalists, social scientists and other media experts. News analysis can also be seen from the lens of complex systems, gathering many types of entities, attributes and interactions over time. As many public figures intervene in different news stories, a first interesting task is to observe the social interactions between these actors. Towards this goal, we propose to use video analysis to automatise the process of constructing social networks directly from news video archives. In this paper we are introducing a system deriving multiple social networks from face detections in news videos. We present preliminary results obtained from analysis of these networks, by monitoring the activity of more than a hundred public figures. We finally use these networks as a support for political studies and we provide an overview of the political landscape presented by the Japanese public broadcaster NHK over a decade of the 7 PM news archives.

**Electronic supplementary material:**

The online version of this article (doi:10.1007/s41109-016-0003-2) contains supplementary material, which is available to authorized users.

## Introduction

In the recent years, the publication of news information has migrated from the traditional means of newspapers, radio, and television to the wider audience offered by the Internet. With the rise of the data-intensive science (Hey et al. 2009) the analysis and monitoring of news information has given birth to the discipline called topic detection and tracking (Allan 2002) which aims at segmenting, identifying, and following information, mainly from raw textual information. News analysis is now going beyond, with image information also being investigated across all varieties of media (Hervé et al. 2013).

The analysis of news information is key to a wide variety of tasks, from sociology and journalism to politics and economy (Cagé 2014). It could help the comprehension of user behavior such as what kind of information a category of users can be exposed to (Resnick et al. 2013). It could also bring new quantitative tools to overcome the limitations of technocratic measures in the investigation of freedom of information (Hazell and Worthy 2010). Even if we know that media competition can lead to a lower quality of information (Cagé 2014), we can hope that public broadcasting services tend to convey an official character, and be a reliable baseline for social analysis.

The contribution of this paper is social networks analysis directly delivered from video content. The social networks are constructed from face detection and tracking of video content from the NHK News 7 broadcast, and enriched with segmentation and domain knowledge. Finally this enables a social analysis of the media representation of politics in NHK over a decade. After discussing related work in the next section, we will present our data in “Face detection and tracking” Section, with characteristics and preprocessing. “Different networks” Section will then introduce the networks we have extracted. We present in “A tool for social analysis” Section social analysis insights of the politico-media landscape of the 12 years of NHK supported by the social networks derived. Because this paper presents preliminary results, we will discuss our observations and future works in “Discussion and future work” Section before concluding.

## Related work

Our system focuses on faces detected in news video. for which new deep learning approaches are very promising: (Schroff et al. 2015) even reaches better-than-human levels of precision in face recognition, although face tracking in videos is not addressed. We use a simpler detection approach inherited from (Viola and Jones 2004) available off-the-shelf but provide face tracking in return.

Many interesting works approach news analysis in a data intensive way, from text analysis. One of the most impressive approach on exploiting news data comes from (Ide and Nack 2013) in which the authors combine news topic threads and demoscopic information to retrieve videos and generate a new summary video to explain prime ministers’ resignations. A Natural Language Processing framework is designed in (Castillo et al. 2013) to characterize news providers, programs, and newsmakers over many channels.

The work from (Sudhahar et al. 2011) is a notable effort in creating networks from news data. They generate *actor-action-object* networks over years of news, with great potential for building narrations and understanding of a news landscape.

News and media have been also material for social studies and especially political studies (Street 2010). Questions such as the media-induced “presidentialization” effect are widely studied (Mughan 2016) and the case of Japan was raised from the Koizumi’s unusual popularity (Krauss and Nyblade 2005). This question is still under investigation (Jou and Endo 2015), but none of these works approach the matter from a network perspective.

The relevance of network modeling for social and political studies does not need to be proven anymore (Lazer 2011), it is actually an old practice (Davis et al. 2009). Beyond classical metrics (Adamic and Glance 2005), networks have been shown to be efficient for topic and concept analysis (Martin et al. 2013), and multiplex networks have been explored to analyse news data (Renoust et al. 2014). In particular, character networks have been broadly analyzed from literature (Waumans et al. 2015), from TV dramas (Nan et al. 2015), and there is even a website dedicated to the social analysis of *Game of Thrones* (Mish 2015).

News data has been one main target for visual analytics applications. Although we do not yet address visualization in this paper, the visual analysis of the networks and timelines was essential to conduct our social analysis. The following examples are all inspiring models to orient our analysis. Visual analysis is brought to help exploring large trans-media news as in (Hervé et al. 2013) and (Itoh et al. 2014) from which not only text but also visual information is used. Faces are also used in the case of (Luo et al. 2007), which fuses many criteria and modalities to support user’s exploration of stories in the corpus, and introduces a network of topics, similarly to (Viaud et al. 2008). Analysis derived from large scale data (Seifert et al. 2014) also includes political figures co-occurrence analysis represented as networks.

## Face detection and tracking

To better interpret the data, we need to draw an accurate picture of what we are looking at. This section details all the preprocessing that is done before computing any social network. After describing the data, we introduce the segmentation of news, the face detection and tracking, and some domain knowledge.

### Description of the data

**Fig. 1:**
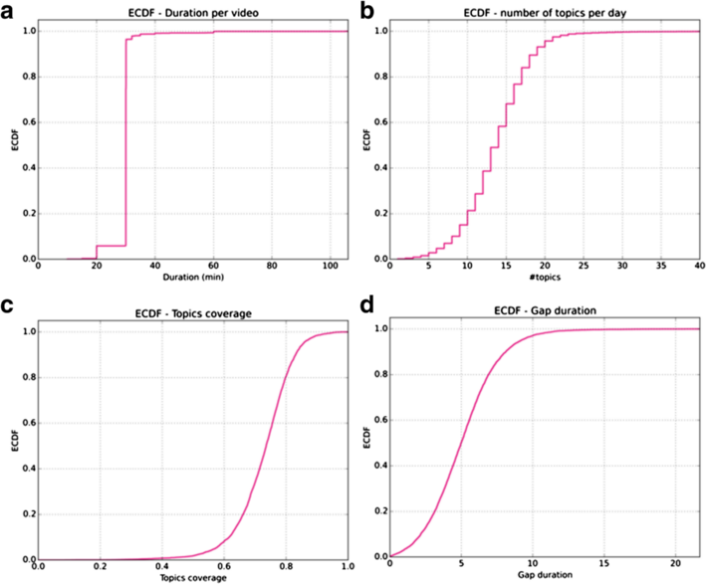
**a** - Most of the programs last 30 min, but some of them may be longer or shorter due to some events like commemorative dates. **b** - We average around 13 topics per day. **c** - Topic segmentation is statistically determined, but most of the news are well covered; only a few topics show large gaps between segmentation (**d**) so we can expect a good overlap with face tracking

Our video dataset consists in the daily-collected NII-TVRECS archive from (Katayama et al. 2005). The capture covers a period between March 17, 2001 and February 27, 2013; of the 4366-day long period, 4259 news programs have been collected cumulating about 2102 h (6.7 TB of video) from the Japanese public NHK channel’s daily News 7 broadcast. The few missing captures concerning mostly the beginning of the time period are due to the capture system setup. Most of the programs usually last 30 mn and only a few of them fall below or beyond this format (news programs may be shorter on Sundays, or longer during commemorative events, see Fig. 1(a)).

### News segmentation

News are specific programs that can be segmented in different news topics. We thank the authors of (Ide et al. 2004) for providing us data in which news topics are derived from textual information (captions) synchronized with the news. In summary, a topic boundary corresponds to a point between sentences where the keyword distribution is distinct between preceding and following windows of sentences.

**Fig. 2:**
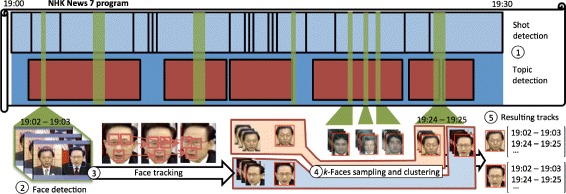
The overall framework of a news video analysis. (1) The video is segmented into topics (*red*) and shots (*blue*). (2) Faces are detected in each frame. (3) Point tracks are inserted and matched across faces creating *face-tracks*. (4) Tracks are sampled and clustered to obtain the final *face-tracks* for each individual (5). Image copyright NHK (Japan Broadcasting Corporation) All rights reserved

As a result, we have a segmentation of the news by topic, based on semantics analysis (segments in *red* in Fig. 2-1). To one *topic* then corresponds what we would commonly call a *news segment* (both terms may be used interchangeably in this paper). Although we do not have the semantic information of these topics (yet), this gives us time boundaries for news segments which will turn useful for analyzing people’s apparition on screen. Overall, taking into account the differences of lengths among programs, this summarizes in a distribution of an average 13.7 news topics per day (*σ*=4.3) as illustrated in Fig. 1(b).

However, this segmentation implies that topics are not consecutively segmented, and gaps may occur between two consecutive topic detections. So topic detections cover on average 72.6 % of the shows (*σ*=0.10), because the beginning (head news summary) and end of the news (weather reports) are ignored by the topic segmentation (Fig. 1(c)). In addition gaps between news segments average 5.1 s (*σ*=2.4) and can stretch up to 20,7 s (see Fig. 1(d)), in which faces may still be detected.

To help the face-tracking process, a standard shot segmentation derived from color histogram thresholding is provided (segments in *blue* in Fig. 2-1). This is a contiguous segmentation, without in-between gaps corresponding to video cut editing.

### Face-track extraction

Now we can extract faces from the video shots as in (Ngo et al. 2013), designed to be applied on large dataset. The extraction is done in the following steps (illustrated in Fig. 2, steps 2 to 5):

- **Detection**. We first apply a detection of faces in all images, using off-the-shelves techniques such as the Viola-Jones face detector (Viola and Jones 2004). Multiple faces can be detected per image, different detections of a same face may also overlap.
- **Tracking**. We now need to group together the detected faces of a same individual into one single face-track. This is done by generating feature points within detected faces – a point track is a same point identified across different consecutive frames. Point tracks from the KLT point tracker (Shi and Tomasi 1994) are matched from a starting face with the following faces given their temporal order and this results in multiple face instances regrouped in face-tracks. There can be multiple face-tracks across the same video.
- **Sampling the face-tracks**. For each face-track, we create a *mean face* that is a representative face in the VGG feature space (Everingham et al. 2006), based on the *k-Faces* method (Ngo et al. 2013). The *mean face* is a mean point in the feature space described by *k* sampled faces.
- **Matching the face-tracks**. Face-tracks can finally be matched based on their *mean face* euclidian distance in the feature space.

The whole process has detected over 30 million faces and 174,778 face-tracks were extracted. We now need to identify and recognize groups of face-tracks, so clustering naturally appears as the following step. However, clustering implies many new issues that we have not yet addressed this work (but we include this goal as part of our future work in “Discussion and future work” Section). Yet, we can still use a different approach to construct our networks, that is of face retrieval.

The faces of 139 characters have been annotated during the evaluation campaign of (Ngo et al. 2013) giving a ground truth for retrieving matching face-tracks. These faces are the faces of well known people among the Japanese media scene, including celebrities and politicians (Japanese and international), for which we had the highest precision of retrieval and identification. In total, over 5 thousands face-tracks were annotated, and 16,714 face-tracks of the 139 different characters were retrieved. This corresponds to 2984 days of news program over the whole archive having matching people, covering a total of 36 h of face tracks.

**Fig. 3:**
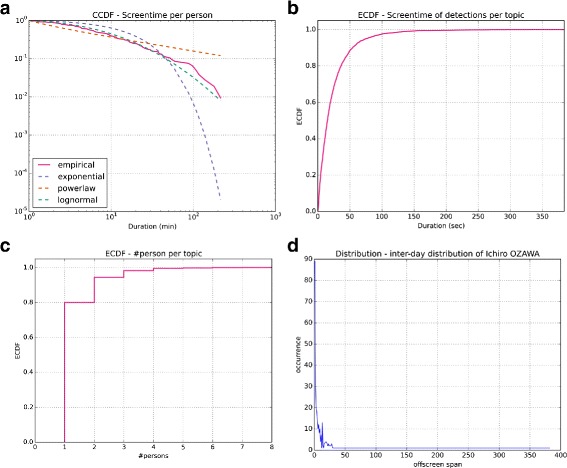
**a** - The distribution of screen time per person fits a log-normal distribution and shows a few people actually hold most of the total screen time. **b** - Face detections in a topic usually average a total of about 24s. **c** - 80 % of the topics detect only one person. **d** - The distribution of time (in days) between two appearances of *I. Ozawa*, which is of one day most of the time, is characteristic of the “bursty” behavior of the data

The coverage of the face-tracks averages 2.4 % of a program (*σ*=2.6), reaching the maximum of 38.9 % of a program. The ground truth has been provided during the 2010 period, for which the face-tracks appear slightly denser (3.3 % on average). The average screen apparition per person is 15.7 min (*σ*=32.5) but there is a lot of variation between people (actually it fits a lognormal distribution Fig. 3(a)), and a few people seem to hold most of the screen time (Table 1).

**Table 1:** The top 10 % people among the different criteria (in bold, the Prime Ministers, and top 5 scores of each criterion). Ichiro Ozawa is the only person taking a top 5 position who has neither been a Prime Minister, nor is a news presenter

Person	Screentime	#Days	#News segments
**Junichiro KOIZUMI**	**215**	**516**	**523**
**Yukio HATOYAMA**	**179**	**330**	**368**
Ichiro OZAWA	**138**	**294**	**304**
**Naoto KAN**	**118**	243	249
Shinichi TAKEDA	**110**	**337**	**491**
**Shinzo ABE**	103	281	**298**
**Yoshihiko NODA**	97	195	245
**Taro ASO**	87	181	187
**Yasuo FUKUDA**	60	144	139
Seiji MAEHARA	55	116	126
Takafumi HORIE	52	116	121
**George BUSH**	45	86	93
Kazuo SHII	42	116	98
Sae NAKARAI	40	**317**	190
Katsuya OKADA	37	115	109
Hideki MATSUI	26	126	73
Wataru ABE	22	84	104

As a result, the tracks work as follows: everyday, we have a news broadcast, and every broadcast contains news segments (topics) and face-tracks of different people. We then observe an average of 23.8 sec of cumulated detections per topics (*σ*=28.9, with a maximum of 383.0 - Fig. 3(b)), with in average 1.28 people detected per topic (*σ*=0.66, with a maximum of 7 - Fig. 3(c)). Thankfully, this shows that we can reasonably expect people to overlap across topics, although 80 % of our news segments do not show more than one person detected (see Fig. 3(c)).

Looking closer at the distribution of inter-day occurrences of people in topics, we can see that most of them appear on screen on a daily basis, with bigger gaps then (Fig. 3(d) is an example). This is sometimes referred as a characteristic of “bursty” data (Wang et al. 2007), meaning that, over the whole period of time, there is a lower probability for two people to be detected together than random, making these links especially interesting. We can also notice that most of the people we are tracking seem to take part in similar topics during the 2008–2011 period.

### About the 139 people

Some background information is necessary for a good understanding of this news data. With a little domain knowledge, we have classified the 139 characters identified into 9 categories depending on what brought them under the light of news: *Politics* (71), *Sports* (27), *Culture* (11), *Business* (7), *Imperial family* (5), *Journalism* (4), *Religion* (3), *Law* (3), and *Other* (5). Additionally, we have enriched them with their country: 96 individuals are from Japan, among which 39 political figures and 22 athletes.

**Fig. 4:**
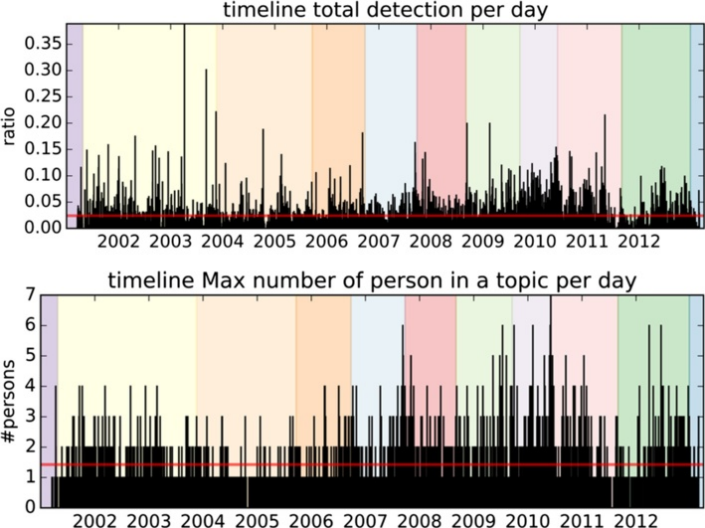
The different time lines for each of the PM reflects well their mandate (as presented in the background colors). Notice the differences in patterns of time apparition for each PM, particularly *Y. Noda* who only appeared during his cabinet

**Fig. 5:**
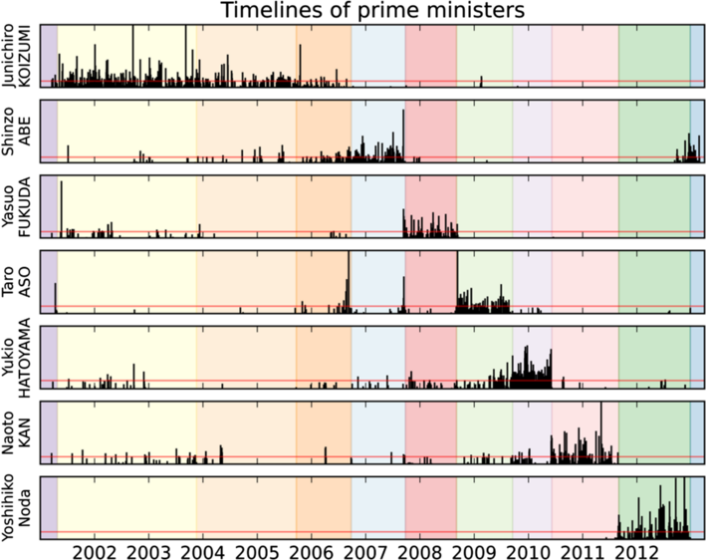
(*Top*) The daily track averages 2.4 % of a program (line in *red*) and shows a bit more coverage during the 2008–2011 period. (*Bottom*) Even if a lot of topics detect one person only, the maximum detections in a topic per day shows many topics going beyond, especially during the 2008–2011 period

Incidentally, the *Politics* class includes 23 international leaders (presidents, prime ministers...). The Japanese prime ministers – hereafter referred as PM – governing during the whole period of capture are of course represented, allowing us to create time frames covering their cabinet(s). *Yoshiro Mori* was the first PM in the timeline, but his mandate only covers a few weeks from the beginning of the capture, so himself is not included in the people subset. Finally, we obtain 11 time periods (Fig. 9 details them in chronological order, note that the numbers following a PM’s name represent different cabinets formed by the same PM). Figures 4 and 5 reflect these different periods as colored section in their background.

**Fig. 6:**
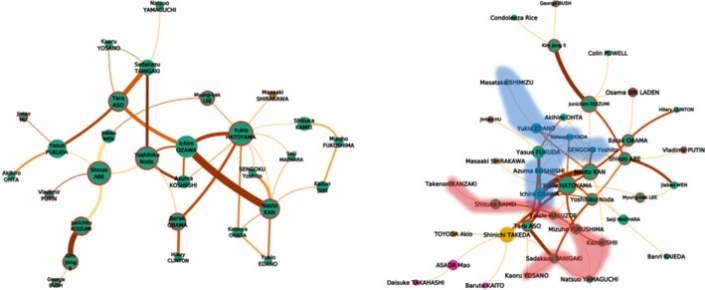
*The networks are better seen zoomed from the bigger version available in Additional file* 1 *: Appendix*. *Green*: politicians,*brown*: businessmen, *yellow*: journalists, *pink*: athletes, *purple*: imperial family. Circled in *red* are world leaders and PM. The size of the node reflects its betweenness centrality. From *light yellow* to *dark orange*, the edges color and width encode their weight. (*Left*) The network of people overlapping on screen. (*Right*) The network of people appearing on a same shot, with two communities in the colored areas

Although Pearson’s correlations between the three measures (*Screentime* *S*, *#days* *D*, and *#news segments* *T* – per person) are very high (*S*−*D*=0.95, *S*−*T*=0.96, and *D*−*T*=0.97), we can use the ranking of the top 10 percentile to extract people of interest (as presented in Table 1).

A background checking gives us supplemental information explaining their occupation of screen space. We can already discard of our analysis the journalists of NHK (*S. Takeda* and *S. Nakarai*) who have an obvious aggregating force due to their role on TV. Out of the obvious known figures and the aforementioned Prime Ministers, *I. Ozawa*, *S. Maehara*, *K. Shii*, and *K. Okada* are famous politicians, and we will talk about them in greater details in “A tool for social analysis” Section. *T. Horie* is a businessman, *H. Matsui* is a baseball player, and *W. Abe* is active on the music scene.

## Different networks

We will now define and present our different networks with their preliminary analysis. We are focusing in this sections on the different ways we can create links of social networks directly derived from the analysis of the video data. Most of the following networks use the people as the same set of nodes, but with different families of ties.

### Network of people overlapping on screen

Our first network connects two people when two face-tracks overlap in time. This means that we create a link between two people when they have been detected *simultaneously* on screen. These links are enriched with the screen duration of the overlapping of tracks as weights.

This network presents 35 nodes and 44 edges, with a main connected component of 29/41 (Fig. 6, left). This connected component is only composed of politicians, with one business person (*M. Shirakawa*, connected to *Y. Hatoyama*). It is worth noting that *J. Koizumi*, the top individual among all other metrics, only presents here a degree of 2. Four nodes stand out in terms of betweenness centrality (*S. Abe:0.16*, *I. Ozawa:0.14*, *Y. Hatoyama:0.18*, and *Y. Noda:0.15*, with the rest of the dataset below 0.09), and 2 nodes in terms of degree (*Y. Hatoyama:10* and *Y. Noda:7*), however no clear convincing cut of communities is shown by Louvain’s algorithm (Blondel et al. 2008).

A few links stand out in terms of screen duration (over 1000), connecting: *Y. Noda* and *S. Tanigaki*, in 2012, *I. Ozawa* and *N. Kan* in 2003, 2006, and 2010, *Y. Hatoyama* and *I. Ozawa* in 2006, 2010, and 2012, *Y. Hatoyama* and *B. Obama* in 2009, *J. Koizumi* and *Kim Jong Il* 2002, *V. Putin* and *S. Abe* in 2012, *T. Aso* and *Y. Fukuda* in 2009. When looking at the number of days in which two different people appear together, we can notice stronger links between: *S. Tanigaki* and *T. Aso* in 2006, *J. Koizumi* and *S. Abe* in 2002, and *H. Clinton* and *B. Obama* in 2008.

### Network of people appearing in a same shot

This second family of ties defines links between people appearing in a same shot (i.e. an uncut segment of video). This network roughly extends the previous network, with the difference that people do not need to appear on screen together. Because shot duration greatly varies depending on the cut of the video, we cannot use it as a meaningful metric to weigh edges, instead, we will consider the number of different days that include these shots.

The network (Fig. 6, center) presents 49 nodes for 75 edges with a main component of 41/71. The maximum *k*-core (*k*=3) (Seidman 1983) presents a very intricate subnetwork of 18 nodes (Fig. 6, right). It includes the PM, and the main anchorman (*S. Takeda*), later referred as the ‘main actors’. All the other nodes are politicians, including *I. Ozawa*. Getting their full list and description may go beyond the scope of this paper, but it is interesting to notice that *N. Yamaguchi* stands out as the only politician not directly connected to any of the PM. The main component presents a wider range of types of people, including 3 athletes, 3 business people, and *O. Bin Laden*. A Louvain segmentation does not present a clear cut of denser subgroups in this network. If we remove the ‘main actors’, we can interestingly observe two communities of politicians (the colored areas in Fig. 6, center), one centered on *M. Fukushima* and *N. Yamaguchi*, and the other one on *K. Okada*. However one should carefully interpret the meaning of these links given the low amount of common shots (at most three).

Three edges stand out with links displaying between 5 and 8 days of connections, *T. Aso* and *S. Tanigaki*, *N. Kan* and *Y. Hatoyama*, *J. Koizumi* and *Kim Jong Il*. If we consider links connecting two people over one day only as ‘casual’ and discard them, we can reveal a network of stronger ties of people with ‘recurrent’ interactions (23/26). In this network, *I. Ozawa* displays the highest betweenness centrality, followed then by the different PM.

### Networks of people appearing during a same news segment

**Fig. 7:**
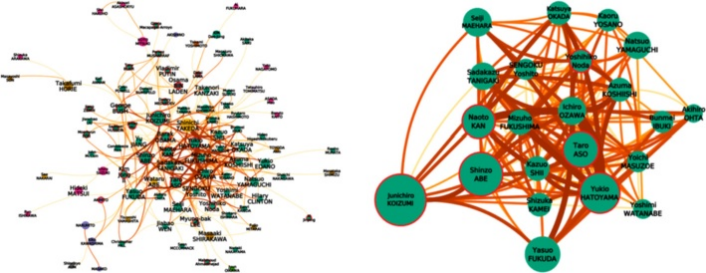
*The networks are better seen zoomed from the bigger version available in Additional file* 1 *: Appendix*. Same encoding as in Fig. 6. Edge weight corresponds to the number of common news segments. (*Left*) The network of people detected during a same topic. (*Right*) The *k*-core (*k*=13) of this network

The following network connects individuals when they have been detected during a same news segment, based on the topic segmentation described in Section 4. This means that two people are connected when they took part of a same media event. The graph connects 107 people over 507 links with a main connected component of 96/499 (Fig. 7, left). This graph presents characteristics closer to complex networks with a long tail distribution of node degrees (actually fitting a lognormal distribution).

Knowing that co-detection during a news segment is the reason linking nodes, we should first remove the journalists – occurring a lot in the dataset, in order to focus on other people’s interactions. The resulting graph presents a maximal *k*-core (*k*=12) gathering 15 Japanese politicians and the 7 PM in a subgraph $G^{\prime }_{k=12}$ with a density $D_{G'_{k=12}}=0.79$ (Fig. 7, right).

**Fig. 8:**
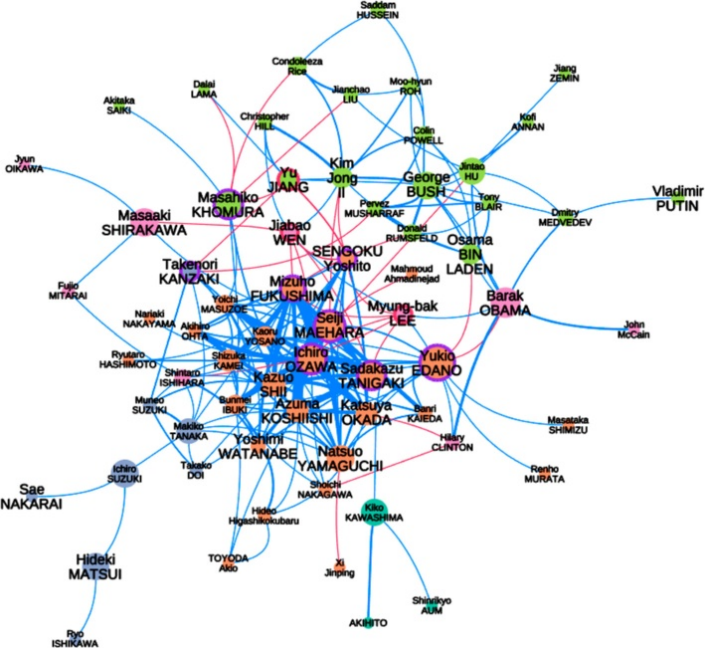
*The networks are better seen zoomed from the bigger version available in Additional file* 1 *: Appendix*. The network derived after filtering nodes from Fig. 7. Edges *width* encode the number of common topics. *Red* edges represent connections between Japanese and foreign politicians (otherwise *blue*). Nodes*color* correspond to the different Louvain clusters, from which we notice the Japanese (center in *orange*) and the international politicians (top in *green*). National politicians with strong ‘foreign’ links are circled in *purple*, and foreign politicians with strong ‘national’ links in *dark red*

A degree and centrality analysis will bring focus to the same people identified in the previous networks. To go beyond, we will look at the graph without the ‘main actors’, leaving 67 nodes for 221 edges. This graph clearly presents community structures, and by running a Louvain algorithm, we obtain a very interesting clustering result. The two main partitions (in *light green*and *orange* in Fig. 8) clearly present international politicians and national politicians (respectively). We are now able to spot the non-PM Japanese politicians who played an active role in international matters by highlighting them (circled in *purple* in the Fig. 8, mostly at the right frontier of the orange community). We do so by counting the number of their ties with international representatives and threshold them based on their cumulative probability distribution (Herman et al. 2000). As a result, we find *Y. Edano*, *S. Tanigaki*, *S. Maehara*, *M. Fukushima*, *Y. Sengoku*, *I. Ozawa*, *T. Kanzaki*, *M. Khomura*. With the same process on the other side, we can identify (circled in red) *Yu Jiang*, *Jiabao Wen*, and *Lee Myung-bak* as having redundant apparition on topics with national politicians. The case of *Lee Myung-Bak* seems to have particularly raised a great interest among national politicians, totalizing 5 connections.

**Fig. 9:**
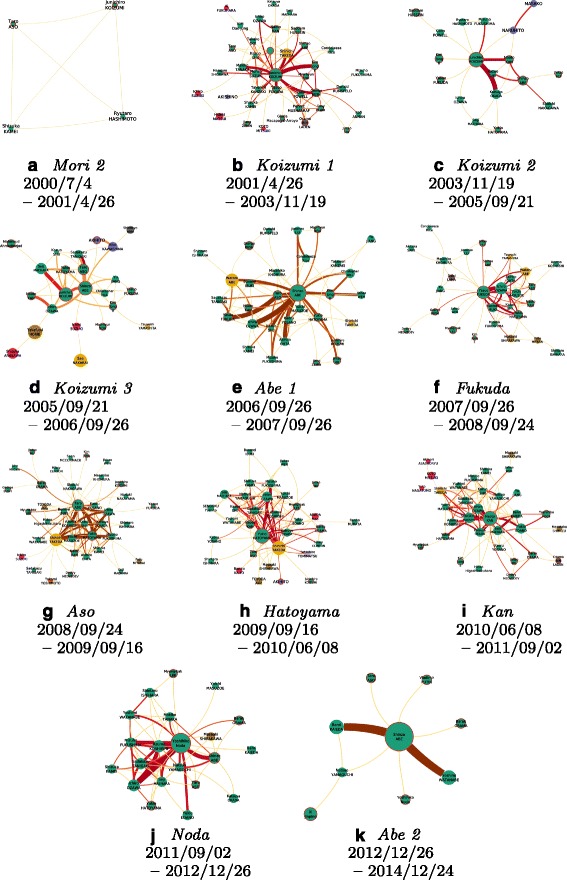
*The networks are better seen zoomed from the bigger version available in Additional file* 1 *: Appendix*. From *Mori 2* (a) to *Abe 2* (k), the topic networks during the different cabinets with the same encoding as in Fig. 6

### Time slicing the common segment network

The topic network has a dynamic multiplex characteristic – although we do not fully exploit this characteristic in this paper. A link is established between two people when matching in different points in time, which means we have virtually an individual link for each matching at different moment in time. Looking at the network in different timeframes will result in different arrangements of the links between nodes and different weights if these multiple links were to be collapsed in one single interaction. Thus, thanks to well defined periods of time corresponding to PM cabinets, we can use topic segmentation as a support to observe not the overall network but each slice involving the people’s interactions over the different cabinets (Fig. 9).

Before we tackle the political analysis in Section 4, we can quickly compare the political landscapes of each cabinet. To do so, we pick out the top 3 Japanese politicians in ranking of centrality and number of news segments, who are neither a PM nor have been detected during the preceding cabinets. In total we have collected 21 prominent politicians, which will be used to compare cabinets one to another. This creates a vector of all politicians per cabinet.

**Fig. 10:**
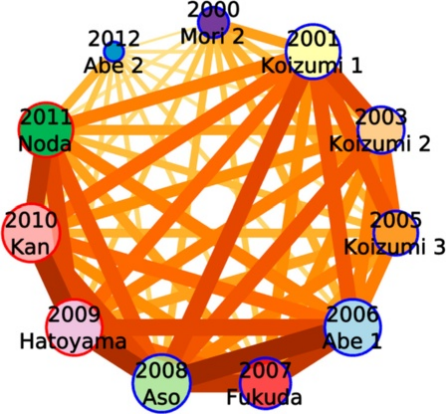
This network shows the (Jaccard) proximity between cabinets given their most visible politicians on the NHK’s scene. The size of nodes encodes the number of people detected during a cabinet. The edges color and size encode the Jaccard proximity (the darker, the closer). The node outlines represent the party in power, *blue* is LDP and *red* is DPJ. We can notice how things have slowly changed from one cabinet to another during the 2006–2011 game of musical PM chairs

Based on these vectors of 21 (+ 7 PM) politicians, we can finally estimate a rough (Jaccard) proximity between cabinets as shown in Fig. 10. The periods from *Abe 1* to *Noda* known for the series of resigning PM, shows the highest proximity one to another, and interestingly to *Koizumi 1*. However, Koizumi’s two following cabinets appear very different, suggesting that he set a very different media/politics scene during this time.

### Preliminary observations

Before we solely focus on a political analysis of the news landscape, the exploration of these networks led us to some understanding of the media/politics scene presented by NHK News 7. Based on this data, together with the knowledge of people, we can confirm that the different PM stand out like no one else in the NHK news. They can be directly identified in all aspects of the data: first, purely quantitatively speaking, they occupy most of the media scene during their own cabinets; then, in the different networks, they also occupy a very central place; the different time-related analysis makes it especially obvious during their cabinets.

We also learn by looking at individual PM: most of them show some level of activity before their mandate and we can observe two opposite cases. On one side, Abe is actually more central than Koizumi himself during *Koizumi 3* (Fig. 9(d)). On the other side, Noda came ‘out of nowhere’ before becoming PM (Fig. 4). Additionally, despite of Hatoyama and Aso appearing quite strong nodes in the different networks, they have never been detected on screen together (Fig. 6) even if they were heading two consecutive cabinets in period of time where the media/politics scene of consecutive cabinets is very similar – maybe because they are the leaders of two opposite parties.

Strikingly, one very particular politician comes out all along this study, *I. Ozawa*, who is (in)famously known as the “Shadow Shogun”. Getting into the details of Ozawa’s role in the Japanese politics is a fascinating work on its own (Meyer 2014), but put in short, after being leader of the opposition, he is known for all the connections and roles he has played behind the scene, building alliances and often changing side – although never he became PM.

Another very interesting point which is worth noting concerns the Imperial family. The Japanese Constitution forbids the Imperial family to take any part in politics, and observing the links surrounding the members of the family are of high interest to survey their actions. Our system finds very little connections (*purple* nodes in Fig. 9(c), (d), and (h)): they mostly concern the revision of the Imperial Household Law because of the issue concerning the succession to the Imperial Throne.

## A tool for social analysis

We want now to investigate this data with the sociologic perspective of studying the political relationships on the media scene over these 12 years. After focusing our data on political activities over time-sliced networks, we introduce political affiliation data, revisit our networks and present our insights.

### Political affiliations

We first need to subset the data: because the *Mori* and *Abe2* cabinets have only partial data, we will discard those from our analysis. We will also focus only on the national and international politically involved actors, reducing to 75 tracked individuals (including 40 Japanese politicians).

**Fig. 11:**
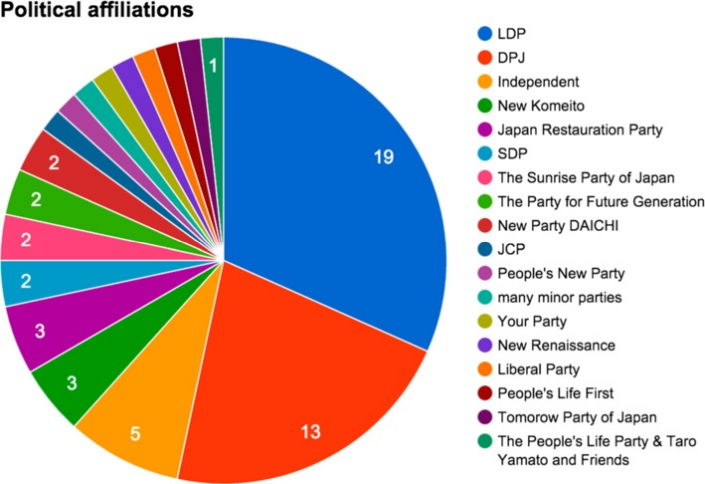
Number of members per political affiliation among politicians in the dataset. Without surprise, DPJ and LDP are draining most of the affiliations

For each of the 40 Japanese politicians, we have manually collected their political affiliations over time. We collected in total 18 different parties (including *independent* politicians and the many *minor parties*) represented among the 40 politicians. As illustrated in Figs. 10 and 11, the two main parties of the Japanese politics are the *Liberal Democratic Party (LDP)* – ruling the cabinets from *Koizumi1* to *Aso* – and its main opposition the *Democratic Party of Japan (DPJ)* – ruling from *Hatoyama* to *Noda*.

As we can see from Fig. 11, the LDP and DPJ accumulate most of the affiliations and many small parties only have one or two members tracked in our dataset. A few politicians have created independent parties at different moments in time. Different coalitions of opposing politicians appeared especially in the end of Noda’s cabinet when it was foreseen that DPJ would not win the next election. This explains why we obtain in total more affiliations than we have politicians.

We are interested in studying how the balance of power reflects in the NHK news. In modern democracies, there are often two main parties: a party in power and its main opposition. In the case of Japan, the leader of the party in power is the Prime Minister, and the leader of the main opposition (hereafter *OL*) is the president of the main party not in power (which is here either DPJ or LDP). However, this leader changes over time such as more than one politician may lead the opposition during a cabinet. To study the opposition, we created an artificial node entitled “*Opposition leader*” (visible in Fig. 13). We extracted from the different persons representing the OL all their detections during their leading mandate and unified them altogether in this abstract entity. This allows us to question the place of the *Opposition leader* in different the political networks.

### Political networks

**Fig. 12:**
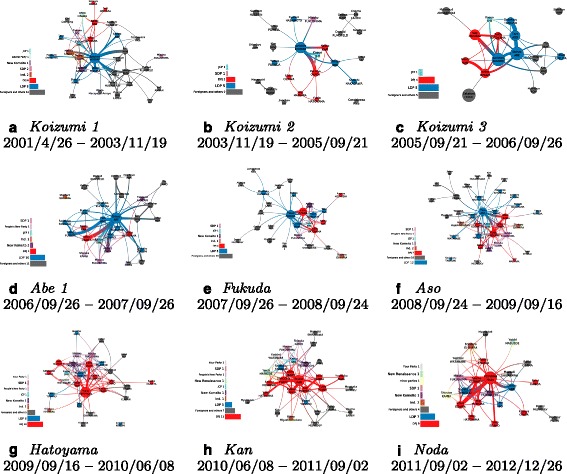
*The networks are better seen zoomed from the bigger version available in Additional file* 1 *: Appendix*. From *Koizumi1* (**a**) to *Noda* (**i**), the networks during the different cabinets colored with the political affiliations. The size of a node (or the width of an edge) encodes the number of news segments in which a (or both) politician(s) appeared

Given this information, we can color the nodes of the previous networks using their political affiliations Fig. 12 – the category *foreigners and others* includes Japanese business people and religious figures who could be involved in political matters. The coverage (number of news segments in which a politician has been mentioned) encodes the size of a node and of a link (for which the co-occurrence is measured). A longitudinal analysis is only permitted using the time sliced topic networks. We can notice that the editorial choice of the NHK seems to well follow the governmental trend, i.e. we detect more LDP members when LDP is ruling, and DPJ members when DPJ is ruling.

**Fig. 13:**
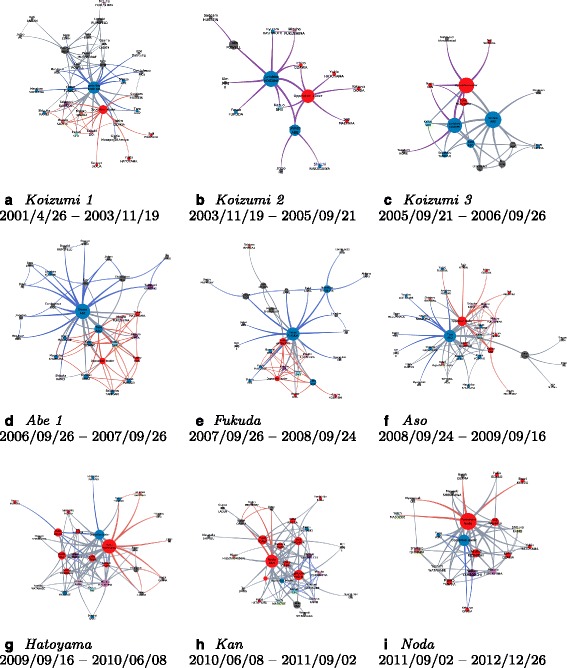
*The networks are better seen zoomed from the bigger version available in Additional file* 1 *: Appendix*. From *Koizumi1* (**a**) to *Noda* (**i**), the networks with the Louvain communities highlighted – red and blue links depending on the PM’s and OL’s affiliation (respectively DPJ and LDP) – purple links if both figure belong to the same community – grey links are out of these communities. The size of a node represents its centrality, nothing encode the size/color of an edge

A quick look at the different networks bring us instantly some interesting insights:

- During *Koizumi1*, foreign affairs appear to show important matters as the PM himself displays many connections with foreign politicians. It is not so much the case during Koizumi’s two following cabinets, in which opposition members appear with more intensity (i.e. more topic coverage as nodes and links). The second cabinet of Koizumi shows a smaller network, but with a strong coverage of Koizumi himself. The third cabinet of Koizumi clearly puts Abe in a stronger position in the network. He shows strong links with opposition figures and even more connections with foreign leaders than the PM himself.
- Abe’s first cabinet shows a real dominance of LDP members with many connections from the PM himself with foreign figures. For the first time, we can notice a rising position of the opposition leader I. Ozawa (and his strong connection with the PM).
- Fukuda’s cabinet also presents a remarkable connection of himself with foreign leaders. M. Khomura is in a central position with foreign affairs (which is expected since he is the Minister of foreign affairs during this period). Foreign affairs appart, we can observe I. Ozawa holding an equally central position as the PM among Japanese politicians.
- The central position of PM Aso during his cabinet cannot be doubted. We can witness many links with foreign leaders, LDP politicians, and the multiple members of the opposition. The opposition gives a large role to DPJ, but is not focused only on I. Ozawa – having a less central position – to the benefit of Y. Hatoyama and some other members.
- Hatoyama’s cabinet bring a clear dominance of the DPJ members, with many other parties represented. I. Ozawa seems to play an important role during this cabinet but the overall topology of this network is denser than the previous one, even in relationship with the foreign figures. This means that the discussion of foreign matters is not focused on the PM, but discussed by all politicians.
- Kan’s cabinet presents similar features than Hatoyama’s with a denser structure between DPJ members, and very limited LDP members (some changed party during this period). Interestingly, there seems to be very limited links between the OL and the PM during this cabinet. We should not forget that Kan’s cabinet has been witnessing the tragic Great East Japan Earthquake and its consequences became the focus of the media scene, rather than the political games.
- Finally, Noda’s cabinet ressembles more of Abe’s or Koizumi’s, with the PM in a very central position. We can only notice that the two main opposition figures (S. Tanigaki and S. Abe) have quite separated positions in the network.

### A small analysis of the political networks

With all uncertainties taken into account (which will be discussed in “Discussion and future work” Section), we want to use the generated networks as support for social analysis. Inspired by recent works in social science (Jou and Endo 2015), general questions drive our study: *How is the opposition represented during the different cabinets?* *Can we find traces of “presidentialization” of the politics in the media?* These questions can of course be tackled quantitatively by studying the coverage of the different actors, but can we learn something else from a social network analysis perspective? To investigate these questions, we propose to observe two phenomena: firstly we can study the place of political leaders in their social networks, secondly we can have a thorough look at the links between the two political parties.

We can then start by focusing the study on the different metrics of the two main nodes (the PM node and the OL node) and their evolution in time (although we will juxtapose different time frames corresponding to cabinets). Relative to their position in the whole network, three quantities especially interest us: their degree centrality (*i.e. how many politician are directly connected to these individuals?*), their betweenness centrality (*i.e. how many people need to go through this politician to access others?*), their closeness centrality (*i.e. how easy is the access of these politicians to the whole network?*). Table 2 summarizes these quantities. We applied the Louvain community detection to each time slice. In most of cabinets, the PM and the OL belong to different partitions (Fig. 13). The size of their respective communities is not a meaningful metric due to the varying size of the networks, but we can notice that both PM and OL reach usually high betweenness centrality in their local community.

**Table 2:** Structural measures of the Prime Minister (PM) and the main Opposition Leader (OL) during the different cabinets

		*Betweenness*	*Degree*	*Closeness*	*Coverage*
Koizumi1	PM	0.172 (1)	25 (1)	0.026 (1)	346 (1)
	OL	0.067 (2)	18 (2)	0.019 (4)	44 (5)
Koizumi2	PM	0.079 (1)	10 (2)	0.048 (1)	118 (1)
	OL	0.051 (2)	11 (1)	0.042 (2)	42 (3)
Koizumi3	PM	0.065 (3)	10 (2)	0.048 (2)	46 (3)
	OL	0.073 (2)	10 (2)	0.043 (3)	36 (4)
	Abe	0.085 (1)	11 (1)	0.050 (1)	48 (2)
Abe1	PM	0.164 (1)	24 (1)	0.033 (1)	150 (1)
	OL	0.011 (4)	10 (3)	0.022 (3)	24 (2)
Fukuda	PM	0.202 (1)	20 (1)	0.030 (1)	75 (1)
	OL	0.008 (8)	10 (2)	0.022 (3)	63 (2)
Aso	PM	0.176 (1)	26 (2)	0.024 (1)	119 (1)
	OL	0.087 (2)	28 (1)	0.020 (2)	88 (2)
Hatoyama	PM	0.099 (1)	22 (1)	0.032 (1)	213 (1)
	OL	0.035 (2)	17 (2)	0.027 (3)	30 (4)
Kan	PM	0.090 (1)	22 (1)	0.029 (1)	150 (1)
	OL	0.003 (12)	11 (6)	0.021 (7)	16 (8)
Noda	PM	0.080 (1)	19 (1)	0.050 (1)	230 (1)
	OL	0.027 (2)	17 (2)	0.040 (2)	56 (2)

*Coverage* is measured in number of news segments

**Fig. 14:**
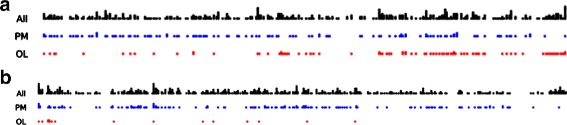
Timelines of links during the cabinets (*Aso* on *top*, *Kan* on *bottom*). We used a “punch card” representation in which the horizontal axis represents the duration of the cabinet from left to right. Each point in the line represents a detection in a news segment, cumulated on the vertical axis. The first line counts all the links in the network, the second the links of the PM, and the third line counts the links of the OL

Although we cannot measure a clear trend such as a growing importance of the OL over time, we can notice a couple of facts. Most often, PM and OL get the top structural positions in the different networks. Except maybe for the PM, more coverage for a politician does not mean higher position in terms of structural measures. We also have two remarkable cases. First is the case of *Koizumi3* in which the OL reaches higher centrality than the PM himself. This is due to the inclusion of a very broadcasted and connected S. Abe who tops all metrics. Then comes the cabinet of Kan: the main opposition does not seems very represented so the OL does not hold an important role. As we mentioned earlier, during this cabinet, NHK was probably more focused on reacting to the tragedy, probably displaying alignment of ideas rather than a diversity of point of views. Figure 14(b) reports the timeline of the news segments attributed to the PM and the OL during Kan’s cabinet, and the last detection of the OL in our dataset only happened a few days before the tragedy.

**Fig. 15:**
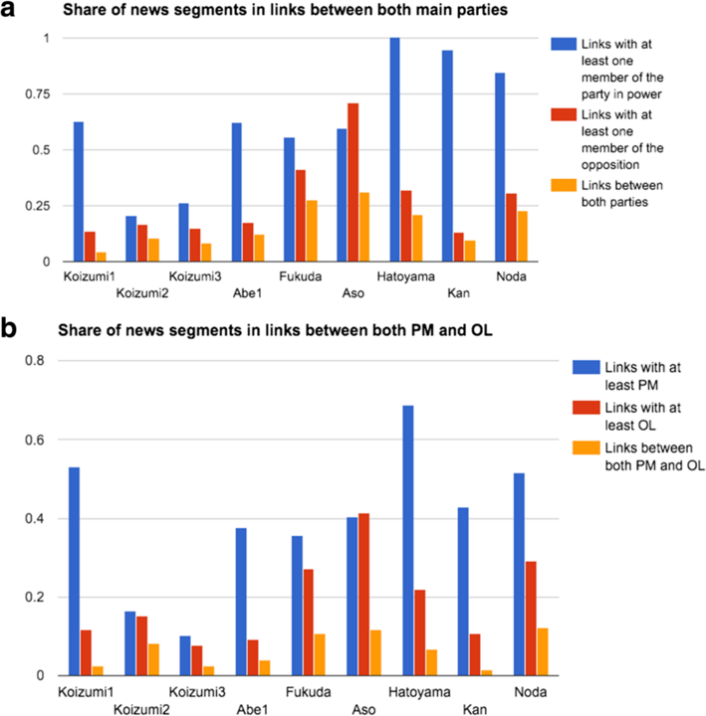
Percentage of links during the different cabinets that: - (**a**) include at least a member of the party in power (*blue*), a member of the main opposing party (*red*), or both (*orange*)- (**b**) include at least the PM (*blue*), the OL (*red*), or both (*orange*)

To study the interactions between the two parties, we can plot the share of news segments that involves each of the two main parties, alongside with the ones that involves both of them together (Fig. 15(a)). It is expected to see most of the cabinets having the interaction of their own members put forward in the media during their own cabinet. This is especially true during all DPJ cabinets, Koizumi and Abe’s first cabinets. Aso’s cabinet displays the opposite behavior. Indeed, it became clear at some point that DPJ will win the following election. At this moment, the media increasingly reported the activity of the opposition (as we can see from the timeline in Fig. 14(a)). This same behavior is confirmed by looking only at the activity of the main figures of both parties (Fig. 15(b)) suggesting that most activities in the political networks are supported by these strong characters.

## Discussion and future work

The different network views provide a powerful tool to understand the media situation, but we also need to draw the limits of the definition of these networks. As for now, the news segment association brings the most meaningful construction of links, even if no actual semantics has yet been introduced into the system. It is equally important to understand how the different pre-processing parts may have strong influences in later interpretations.

**Fig. 16:**
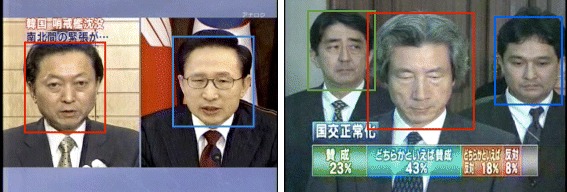
The difference between screen co-occurence on a ‘split screen’ (*Left*) or within a same picture (*Right*) – Image copyright NHK (Japan Broadcasting Corporation) All rights reserved

The screen overlap network has the strongest family of links in terms of social ties, but it is also the most subject to controversy in two ways. First, because of the bursty characteristics of our data, the limited but reliable subset of people, and parameters of our face detectior make the amount of screen co-detections limited. Then, because many detections concern split screens, which in turn often means an opposition of ideas on a same subject, hence defining a sort of *negative* link – something we would like to investigate in the future. We want to distinguish this case from the screen co-occurence, which holds the different meaning of people standing in the same room at the same time (Fig. 16).

The same comment may also be made on the shot co-occurrence network, which finally extends the latter with a lighter meaning. For example, some shots occur behind the anchorman switching from one segment to another, sometimes leading to false positive links. Besides the system showing a good accuracy (Ngo et al. 2013), some face occurrences may remain untracked, but we can still draw our conclusions thanks to the large period of time we observe.

This leads us to discuss the effectiveness of our system. Our contribution solely focuses on face-tracking because we have a long-term goal to use the screen time as a reliable measure. Our current experiment shows that measuring screen time is yet limited, and we need to discuss the accuracy of our system to understand this limitation. Our face-tracking reaches about 61 % precision on the NII-TVRECS dataset (Katayama et al. 2005), for which we estimate an average of 37 % recall (Nguyen et al. 2010; Ngo et al. 2013). This measures the quality of the *retrieval* of people’s face-tracks, keeping in mind that we had performance constraints so we could applied face-tracking on the whole database. Because we are focusing on annotated face-tracks, we made sure that the subset we are exploring for this paper reach 100 % precision. However, our system still misses a lot of face-tracks due to our low recall.

The recall depends on the multiple steps described in the first part of this document.

- It first depends on the performances of our face detector, the Viola-Jones detector (Viola and Jones 2004), which performs up to 88 % accuracy depending on conditions and parameters. We chose this detector because of it is rather fast to execute (on our large database) and it is readily available from openCV (Bradski and et al. 2000). The main drawbacks of this detector are its sensibility to posture and lighting conditions, and the high number of false positive among small size faces (so we actually set a minimum face size of 60×60 pixels). Nowadays, deep learning approaches such as in (Farfade et al. 2015) reaches over 97 % of accuracy in detection, with far less sensibility to orientation and lighting.
- Our tracking phase also filters out some detected faces because we use a point tracker, which, by definition, can be very sensible to lighting conditions and occlusions (although we made efforts to improve its robustness (Ngo et al. 2013)). Indeed, we detected some face instances without reliable tracking, so we could not keep them as face-tracks and discarded them. Optical flow approaches, such as in (Ranftl et al. 2015), seem to be very promising improvements.
- The last step concerns of course the face-track recognition, for which we apply a *k*-Faces sampling (Ngo et al. 2013) to match *mean faces* described using VGG features (Everingham et al. 2006). Once more, deep learning approaches, such as in (Schroff et al. 2015) and (Taigman et al. 2014), bring higher-than-human performances (over 97 % of accuracy). We can expect excellent results by matching *mean faces* using these deep features, and at least retrieve more face-tracks of a same individual.

Additionally, the networks we exploited to support social analysis were derived from news segment associations, i.e. networks built when two individuals appeared in a same news segment. One can argue that we would reach much higher accuracy by textual information instead of visual information. Indeed, named entity recognizers (NER) can reach high recall, sometime over 90 % for the Stanford NER (Finkel et al. 2005) on “Person” detection (see (Atdag and Labatut 2013) for comparisons). This depends of course of the dataset, training, and above all, language models. Although results could reach up to 62 % accuracy for “Person” detection in Japanese (Ichihara et al. 2015), we want to keep in mind that actually *seeing* somebody on TV and *hearing* a person’s mention has a different impact on viewers. However, we will definitely keep the analysis of the textual source and construction of networks from mentions as a future work. The comparison between both sources of information will actually be even more interesting.

Since we are discussing the data itself, our future work will extend the set of people to all faces detected in the dataset, not limited to the tagged individuals. We also put effort in enhancing the recall and the precision of the detection; and the addition of semantic information derived from the topic detection will be a great improvement.

This paper only scratches the surface, but the analysis of news data craves for application of many network analysis techniques. For example, the different families of links (screen, shot, segments, semantics, parties, etc.) also form a multiplex network as in (Kivelä et al. 2014), for which we can search for metrics (Battiston et al. 2014). We can draw multiplex networks as in (Renoust et al. 2014) with people interacting through cabinets and hopefully find cohesive groups of politicians. The dynamic of links is also of great interest and *Δ*-cliques (Viard et al. 2016) (cliques over time in a stream of links) is a promising lead. In addition to finding outliers, we will be interested in groups of political actors who regularly appear together among similar subjects.

As for the social analysis, these networks provide a fertile ground to completely investigate political questions. The definition of the links is extremely important though. To provide convincing conclusive analysis, links and weights need to be clearly interpreted - the unambiguous non-split screen co-occurrence being the best. Using the screen co-occurrence will require to measure exactly the uncertainty of the detection and of the matching, so screen-time may be turned into a reliable variable for regression.

We have not studied the diversity of political opposition, but there are many other parameters we would like to investigate: for example, we could create meta-networks of political parties and observe who bridges them and when do these links occur, we could also focus on the role of the opposition with foreign leaders. We believe that such analysis will ultimately bring quantitative evidence of social phenomenon, such as the “presidentialization” of the Japanese politics (Jou and Endo 2015).

## Conclusion

This work has introduced the production and analysis of face detection and tracking data over twelve years of news broadcast. We have detailed the data’s characteristics and brought a few outliers. Together with the use of topic segmentation and limited domain knowledge, we have derived many networks, each presenting a different point of view on the data, conducting to the understanding of the politico-media situation displayed by NHK during this period of time.

The combined views of these networks show interesting insights on the story behind the data, an arguably clear picture of the media/politics landscape during the different cabinets, also isolating key players at different levels. That is what the general reader may take away: thanks to the networks, even those knowing nothing of the Japanese media landscape can quickly get an idea of who the main actors are, and their relative importance. Of course, the level of comprehension will increase as we improve the precision of our detectors, and the semantics of our links.

We have demonstrated in this small use case an entire pipeline: how – from raw video data – we can create networks that can tell us a story of the politico-media scene in Japan, with the help of their structural properties. Of course, to support a full political analysis, we need to assess the uncertainty of links derived from the face detection, since we want to test hypothesis with these tools.

Rather different to the classical topic detection and tracking approaches of news data, this work brings up if not confirms the relevance of network analysis derived from news data. By itself, this is also an interesting framework for many potential contributions to the current challenges of social network analysis – including, but not limited to, multiplex and multi-attributed network analysis, dynamic networks, and their combination.

Finally, this work has given us useful directions that will help us design visual analytics tools, which we wish to put in the hands of domain experts, sociologists and journalists, to conduct the in-depth analysis of over 12 years of news.

To ease the reading flow of the paper, we propose a larger format of all small figures in the Additional file 1: Appendix section at the end of this document. Additionally, note that all figures are zoomable on the digital copy of the document.

## Additional file

Additional file 1 Appendix. (PDF 5520 kb)
