# Supplementary material for: When face-tracking meets social networks: a story of politics in news videos
Source: Appl Netw Sci. 2016 Jun 1;1(1):4. doi: 10.1007/s41109-016-0003-2 (PMC6245148; doi:10.1007/s41109-016-0003-2)
Supplement: Supplementary file 1 — Appendix. (PDF 5520 kb) [file 41109_2016_3_MOESM1_ESM.pdf]

Appendix

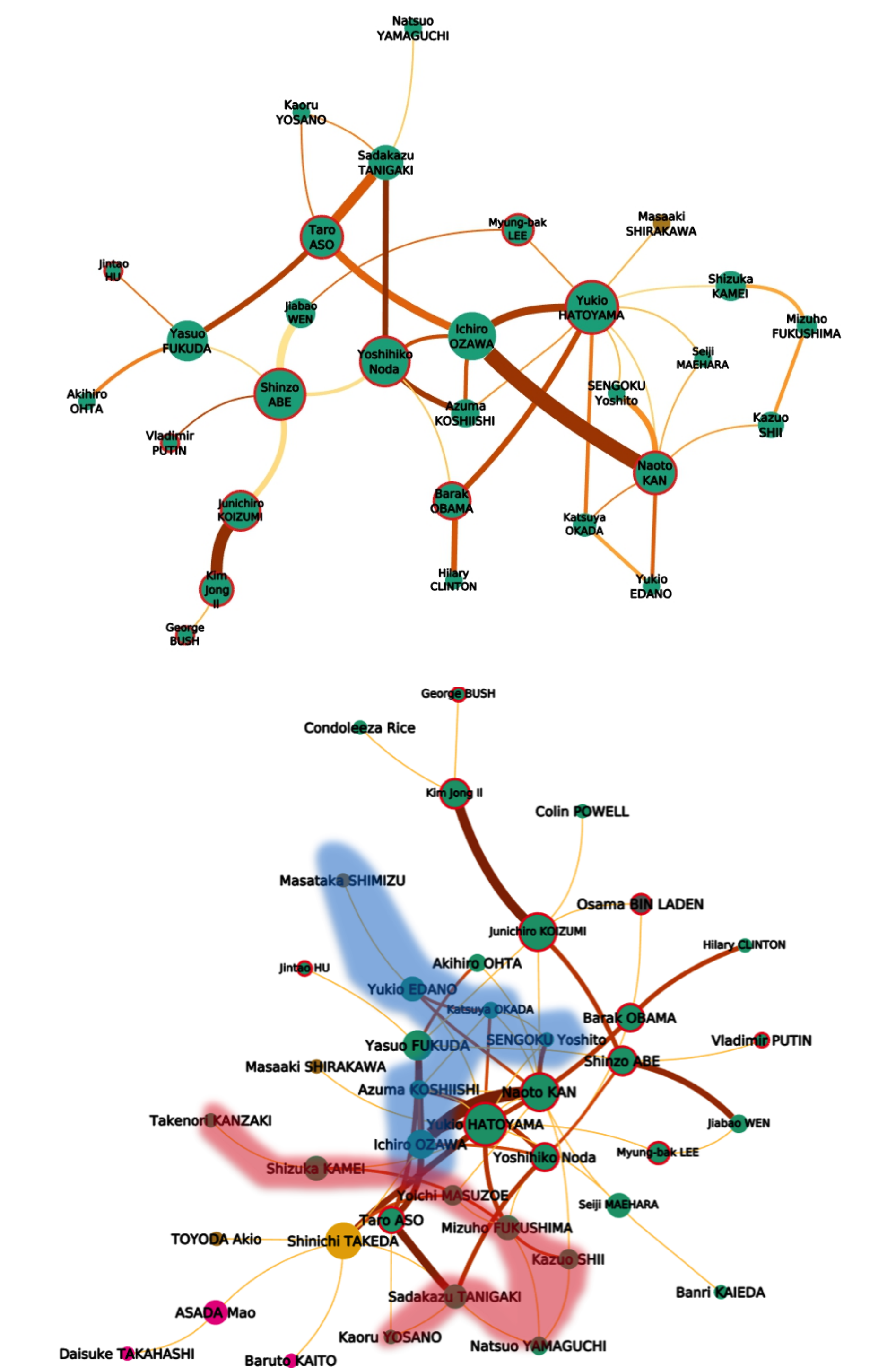

Figure 6 *The pictures better seen zoomed*. *Green*: politicians,*brown*: businessmen, *yellow*: journalists, *pink*: athletes, *purple*: imperial family. Circled in red are world leaders and PM. The size of the node reflects its betweenness centrality. From light yellow to dark orange, the edges color and width encode their weight. (*Top*) The network of persons overlapping on screen. (*Bottom*) The network of persons appearing on a same shot, with two communities in the colored areas.

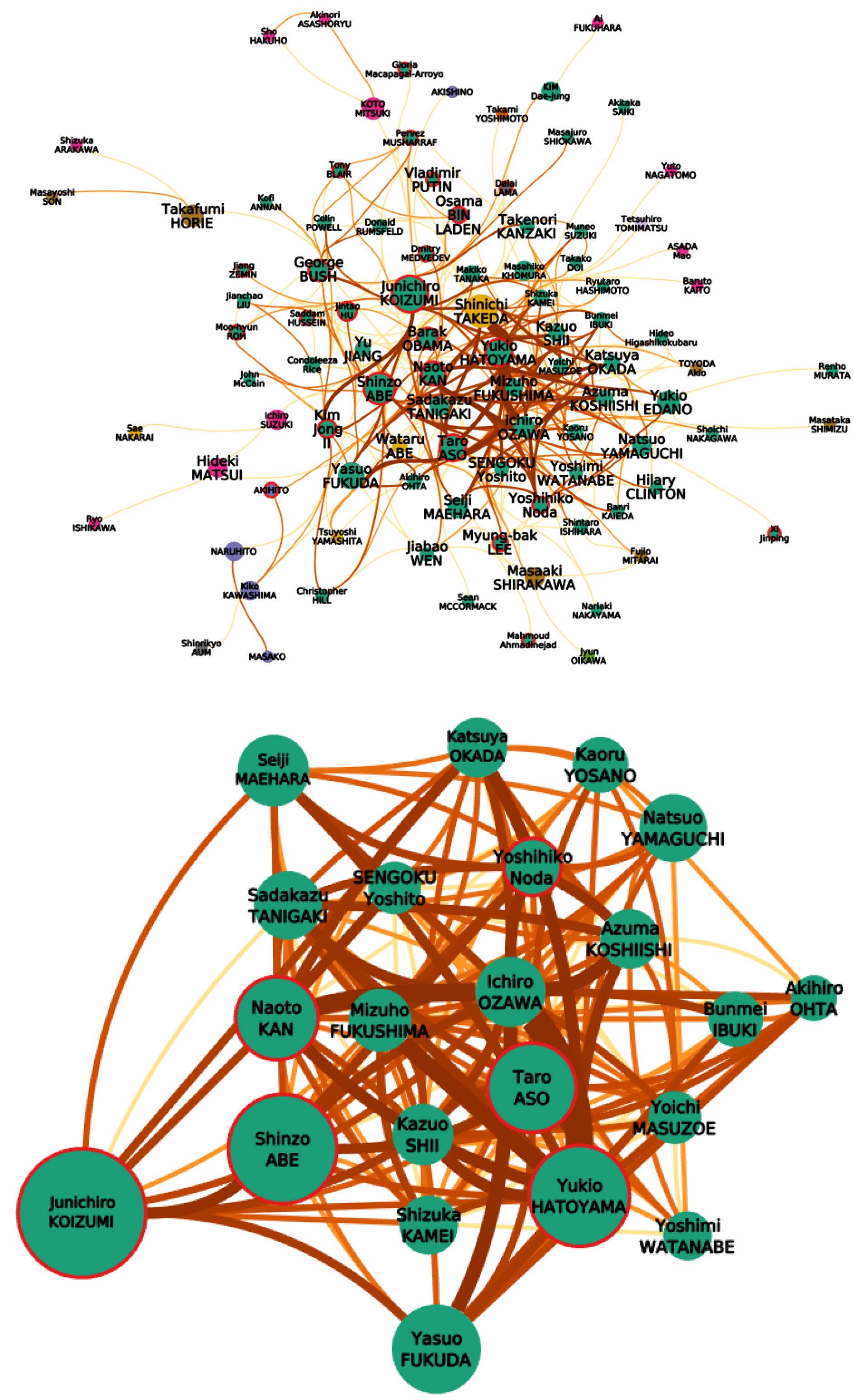

Figure 7 *Pictures better seen zoomed.* Same encoding as in Figure 6. Edge weight corresponds to the number of common news segments. (*Top*) The network of persons detected during a same topic. (*Bottom*) The k-core ($k = 13$) of this network.

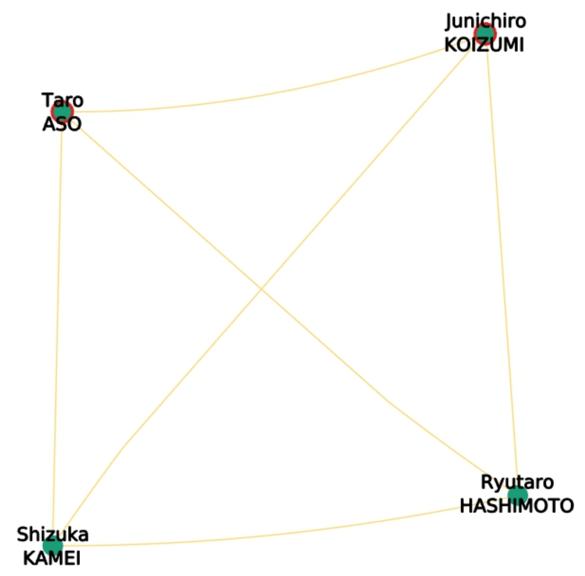

(a) *Mori 2: 2000/7/4 – 2001/4/26*

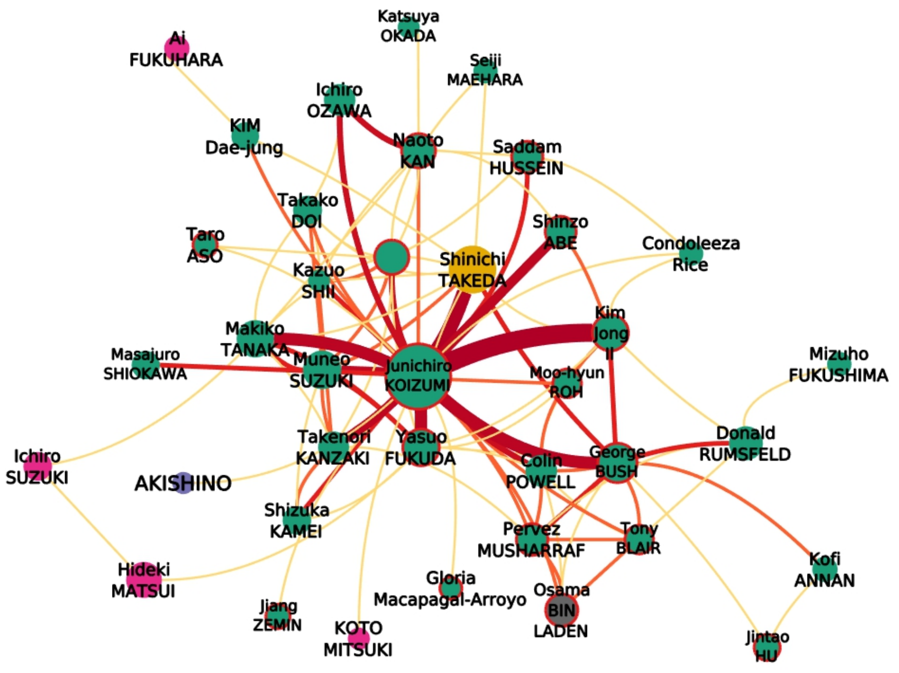

(b) *Koizumi 1: 2001/4/26 – 2003/11/19*

Figure 9 *The networks are better seen zoomed*. From *Mori 2* (a) to *Abe 2* (k), the topic networks during the different cabinets with the same encoding as in Figure 6.

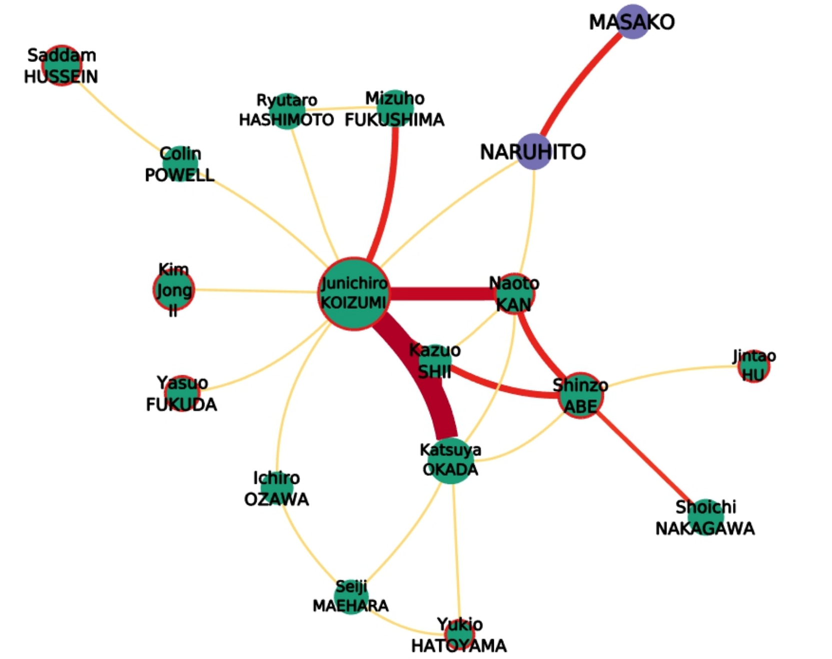

(c) *Koizumi 2: 2003/11/19 – 2005/09/21*

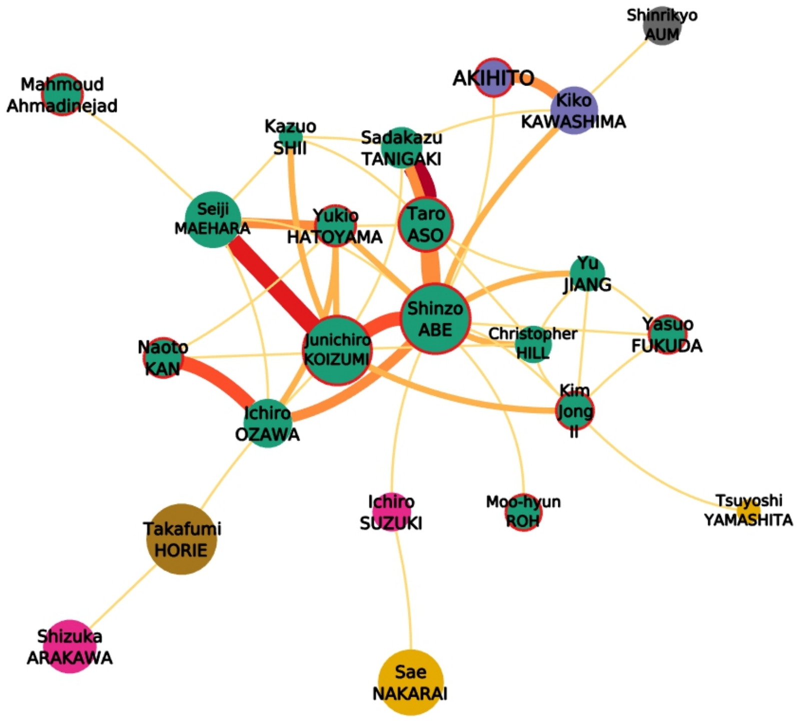

(d) *Koizumi 3: 2005/09/21 – 2006/09/26*

Figure 9 *The networks are better seen zoomed*. From *Mori 2* (a) to *Abe 2* (k), the topic networks during the different cabinets with the same encoding as in Figure 6.

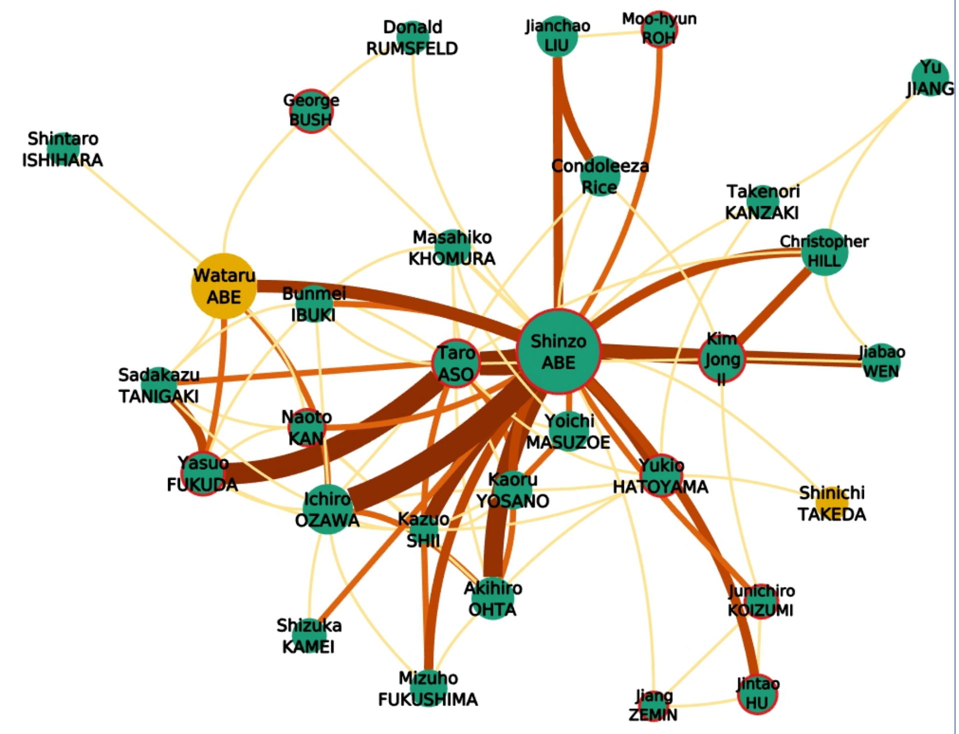

(e) *Abe 1: 2006/09/26 – 2007/09/26*

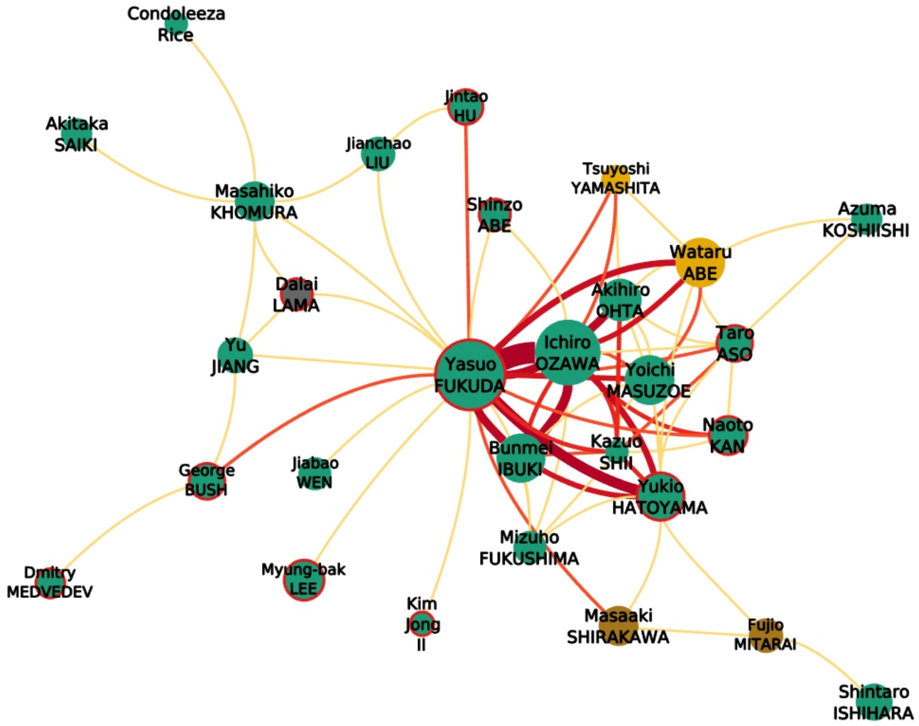

(f) *Fukuda: 2007/09/26 – 2008/09/24*

Figure 9 *The networks are better seen zoomed*. From *Mori 2* (a) to *Abe 2* (k), the topic networks during the different cabinets with the same encoding as in Figure 6.

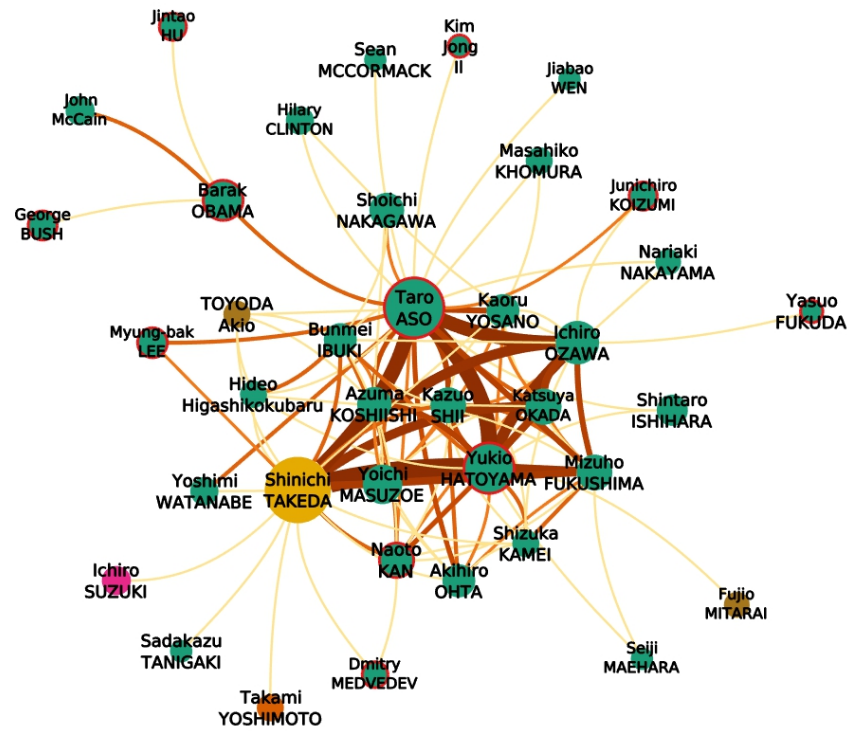

(g) *Aso: 2008/09/24 – 2009/09/16*

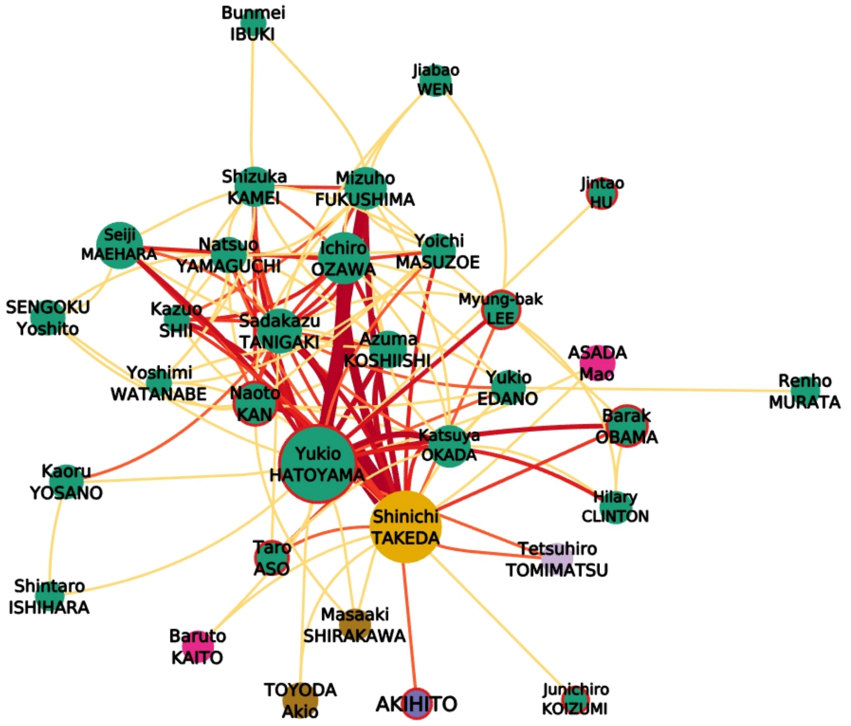

(h) *Hatoyama: 2009/09/16 – 2010/06/08*

Figure 9 *The networks are better seen zoomed*. From *Mori 2* (a) to *Abe 2* (k), the topic networks during the different cabinets with the same encoding as in Figure 6.

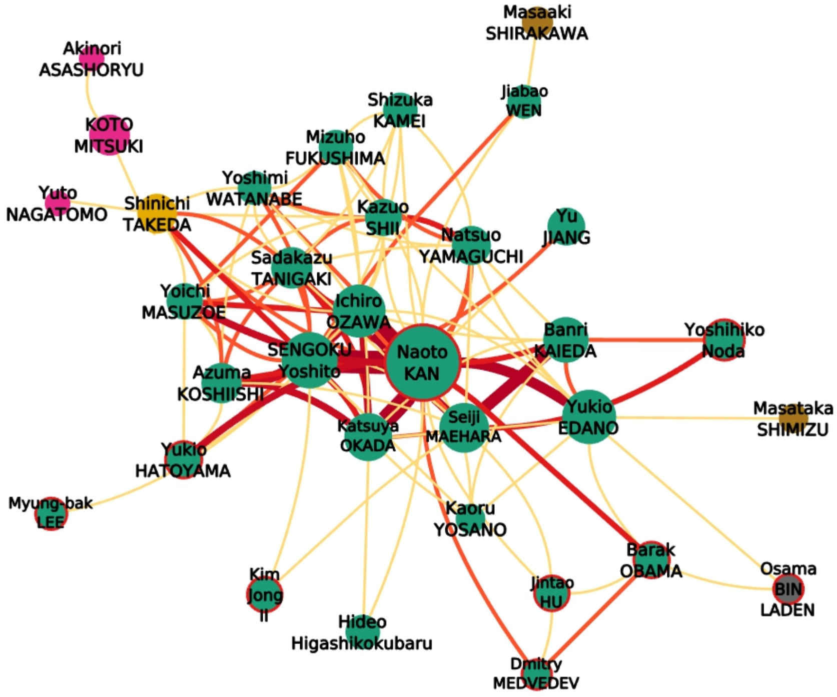

(i) *Kan: 2010/06/08 – 2011/09/02*

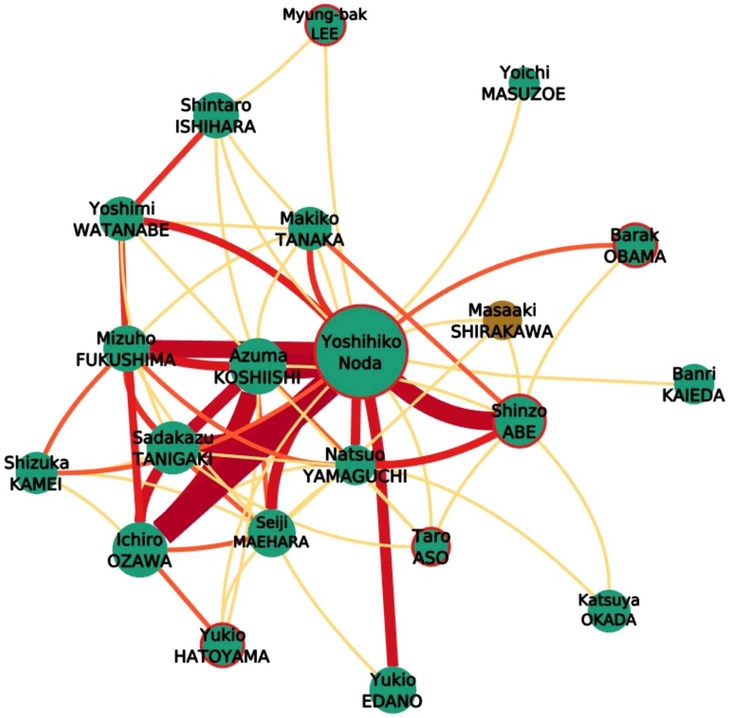

(j) *Noda: 2011/09/02 – 2012/12/26*

Figure 9 *The networks are better seen zoomed.* From *Mori 2* (a) to *Abe 2* (k), the topic networks during the different cabinets with the same encoding as in Figure 6.

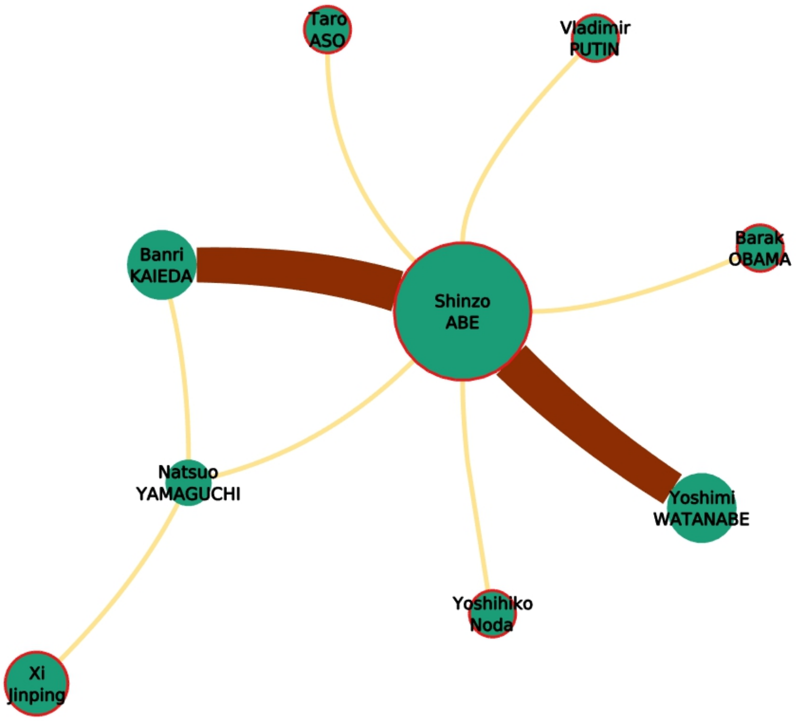

(k) *Abe 2: 2012/12/26 – 2014/12/24*

Figure 9 *The networks are better seen zoomed*. From *Mori 2* (a) to *Abe 2* (k), the topic networks during the different cabinets with the same encoding as in Figure 6.

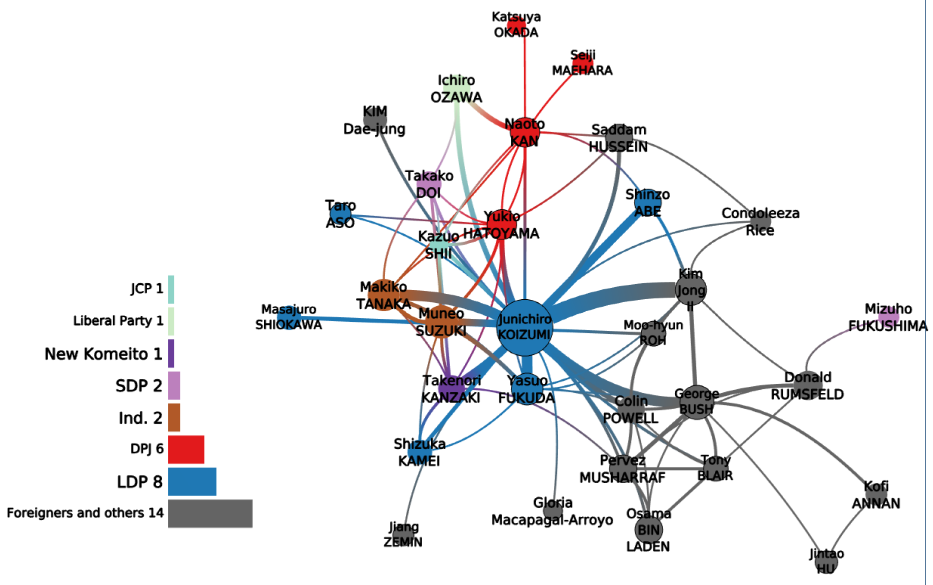

(a) *Koizumi 1: 2001/4/26 – 2003/11/19*

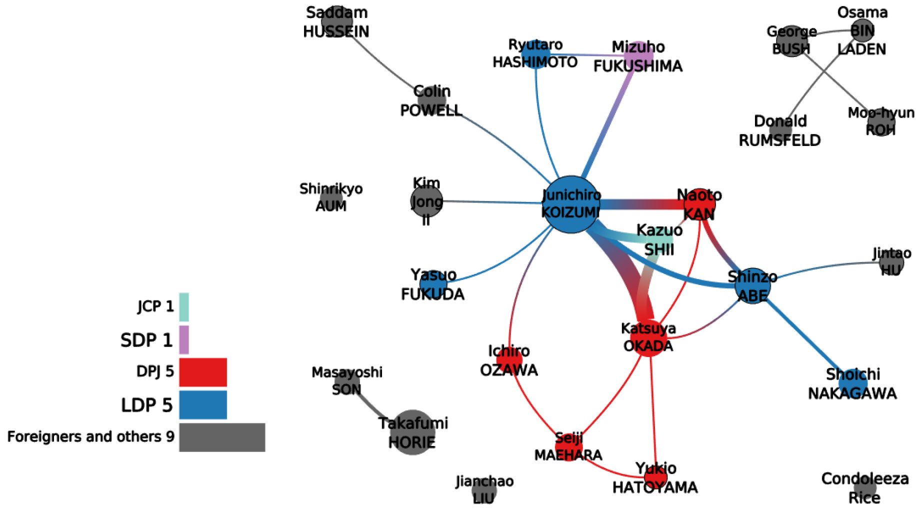

(b) *Koizumi 2: 2003/11/19 – 2005/09/21*

Figure 12 *The networks are better seen zoomed*. From *Koizumi1* (a) to *Noda* (i), the networks during the different cabinets colored with the political affiliations. The size of a node (or the width of an edge) encodes the number of news segments in which a (or both) politician(s) appeared.

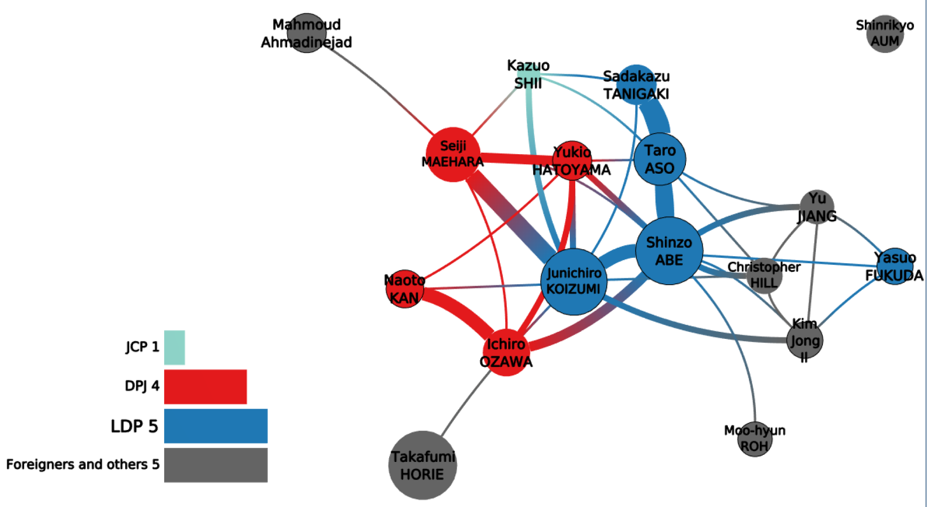

(c) *Koizumi 3: 2005/09/21 – 2006/09/26*

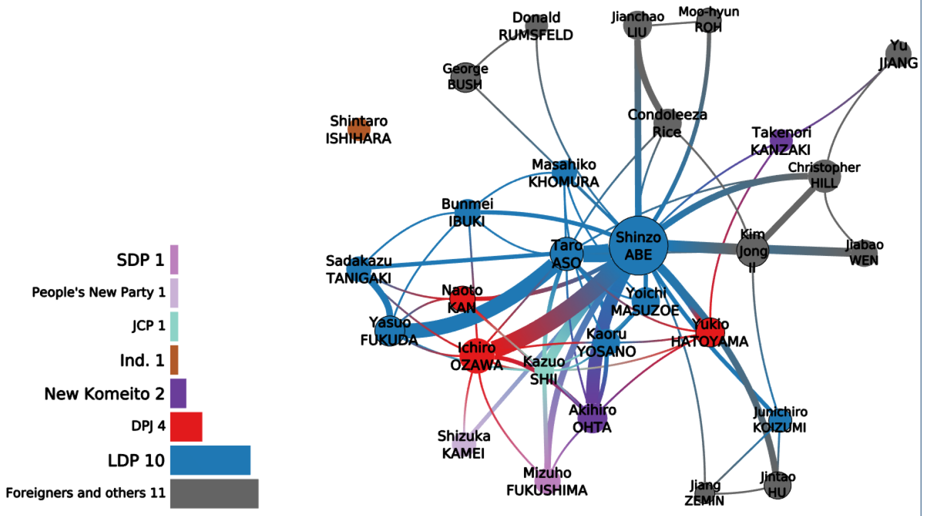

(d) *Abe 1: 2006/09/26 – 2007/09/26*

Figure 12 *The networks are better seen zoomed*. From *Koizumi1* (a) to *Noda* (i), the networks during the different cabinets colored with the political affiliations. The size of a node (or the width of an edge) encodes the number of news segments in which a (or both) politician(s) appeared.

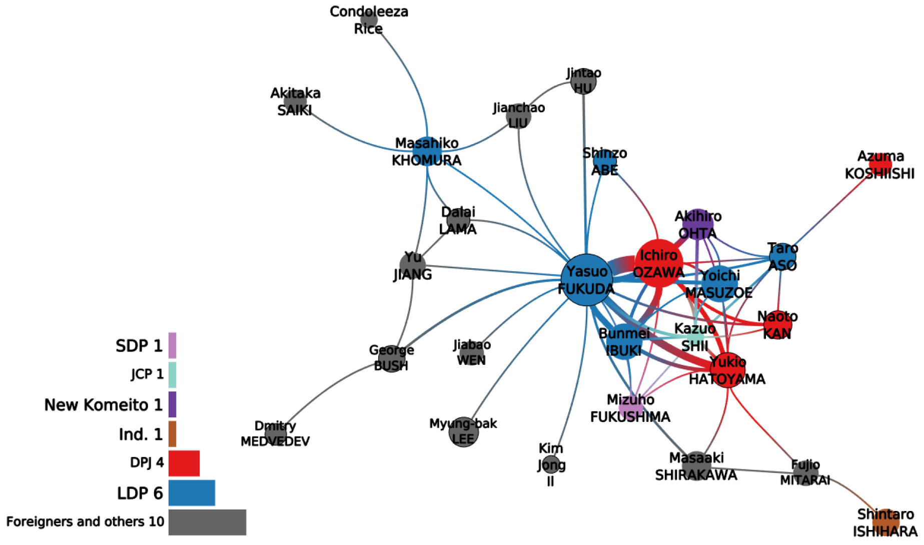

(e) *Fukuda: 2007/09/26 – 2008/09/24*

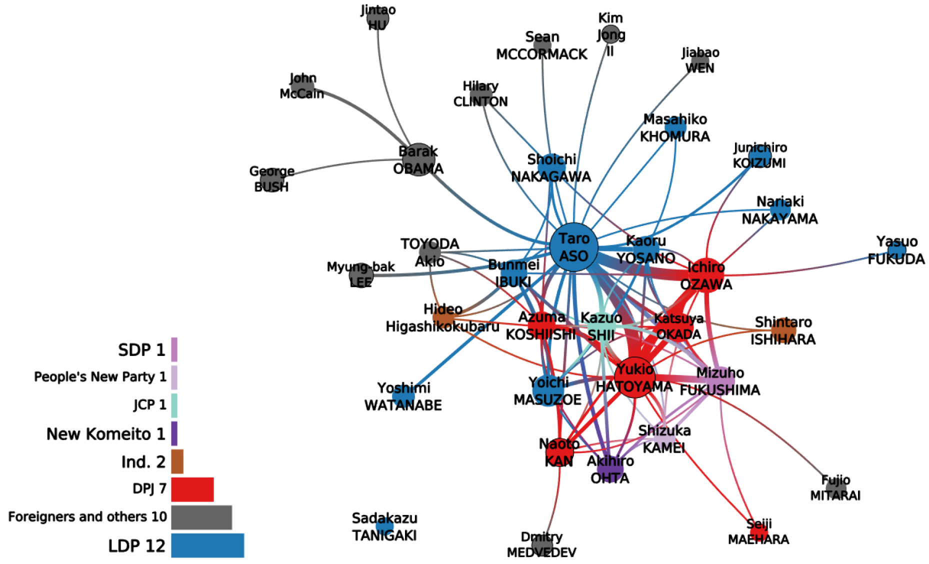

(f) *Aso: 2008/09/24 – 2009/09/16*

Figure 12 *The networks are better seen zoomed*. From *Koizumi1* (a) to *Noda* (i), the networks during the different cabinets colored with the political affiliations. The size of a node (or the width of an edge) encodes the number of news segments in which a (or both) politician(s) appeared.

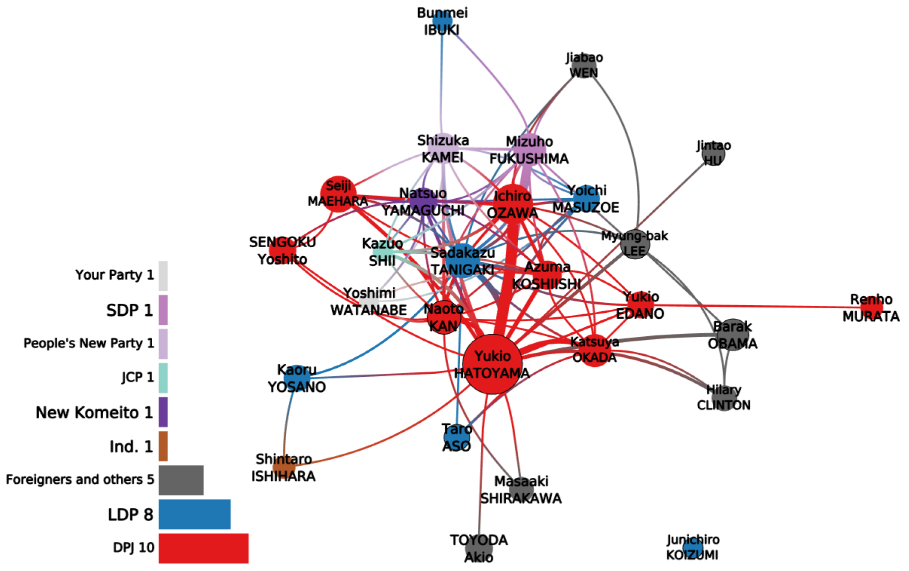

(g) *Hatoyama: 2009/09/16 – 2010/06/08*

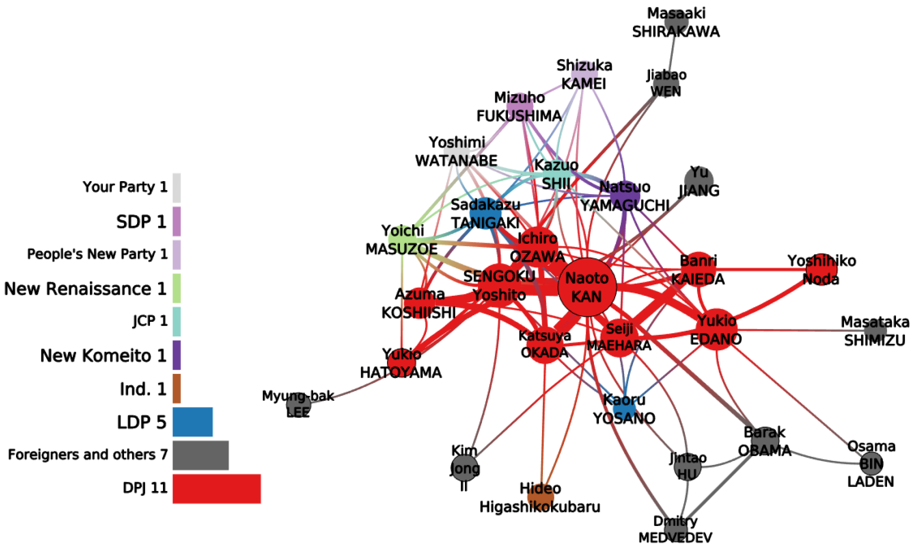

(h) *Kan: 2010/06/08 – 2011/09/02*

Figure 12 *The networks are better seen zoomed*. From *Koizumi1* (a) to *Noda* (i), the networks during the different cabinets colored with the political affiliations. The size of a node (or the width of an edge) encodes the number of news segments in which a (or both) politician(s) appeared.

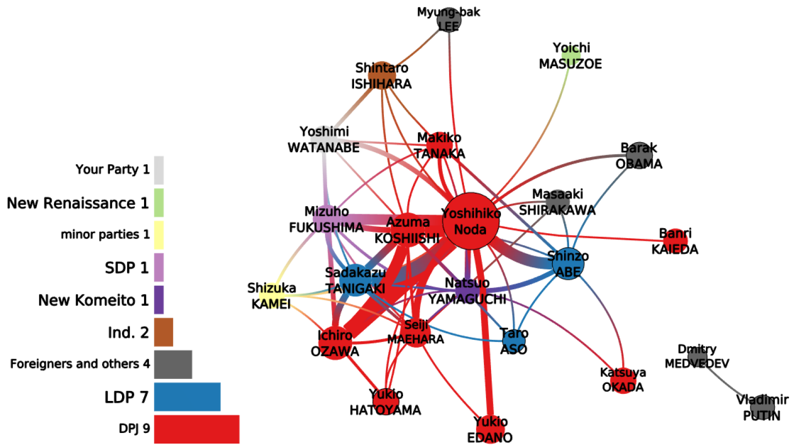

(i) *Noda: 2011/09/02 – 2012/12/26*

Figure 12 *The networks are better seen zoomed*. From *Koizumi1* (a) to *Noda* (i), the networks during the different cabinets colored with the political affiliations. The size of a node (or the width of an edge) encodes the number of news segments in which a (or both) politician(s) appeared.

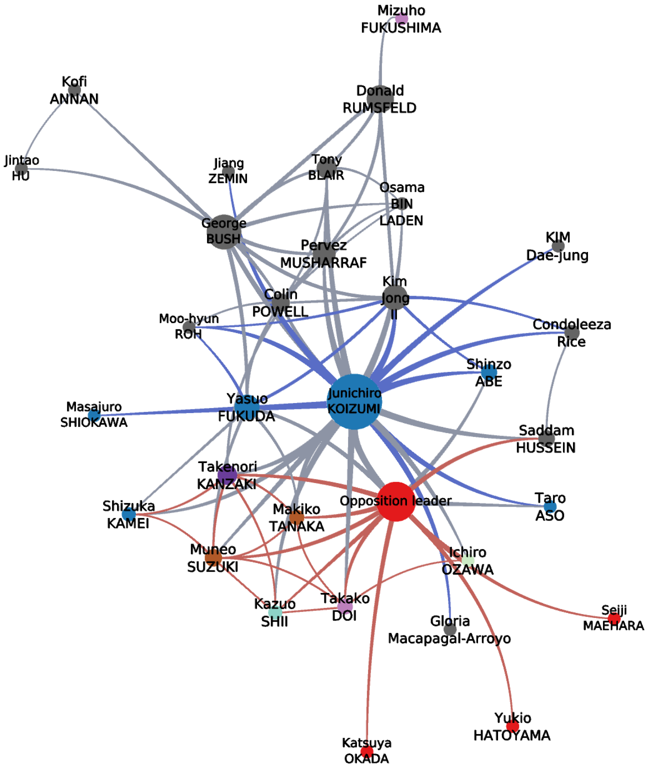

(a) *Koizumi 1: 2001/4/26 – 2003/11/19*

Figure 13 *The networks are better seen zoomed*. From *Koizumi1* (a) to *Noda* (i), the networks with the Louvain communities highlighted – red and blue links depending on the PM's and OL's affiliation (respectively DPJ and LDP) – purple links if both figure belong to the same community – grey links are out of these communities. The size of a node represents its centrality, nothing encode the size/color of an edge.

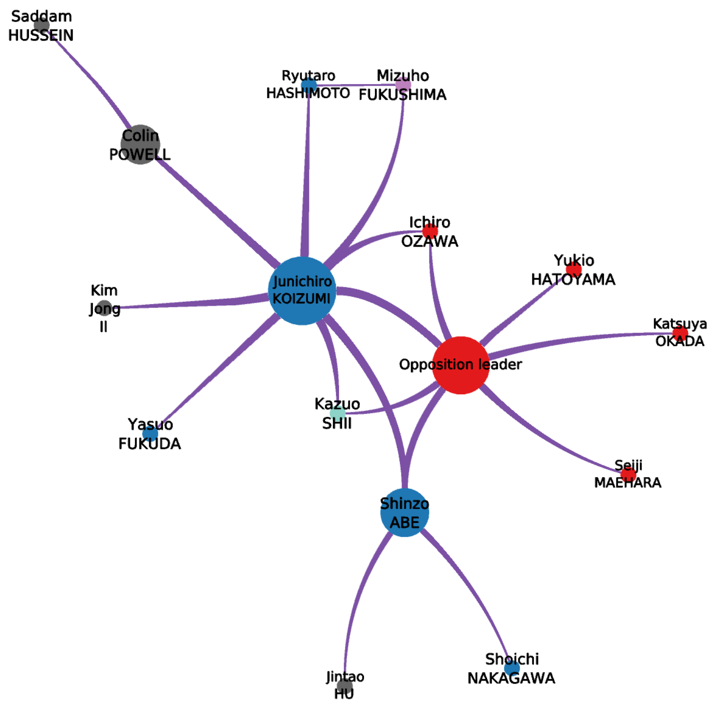

(b) *Koizumi 2: 2003/11/19 – 2005/09/21*

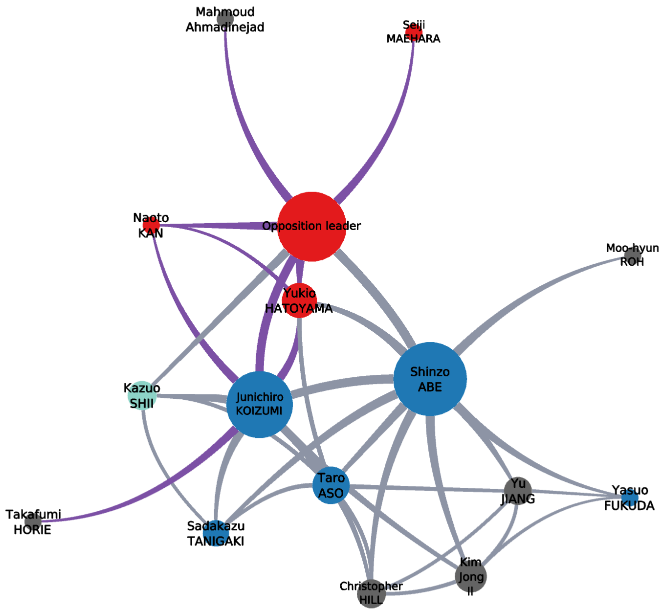

(c) *Koizumi 3: 2005/09/21 – 2006/09/26*

Figure 13 *The networks are better seen zoomed*. From *Koizumi1* (a) to *Noda* (i), the networks with the Louvain communities highlighted – red and blue links depending on the PM's and OL's affiliation (respectively DPJ and LDP) – purple links if both figure belong to the same community – grey links are out of these communities. The size of a node represents its centrality, nothing encode the size/color of an edge.

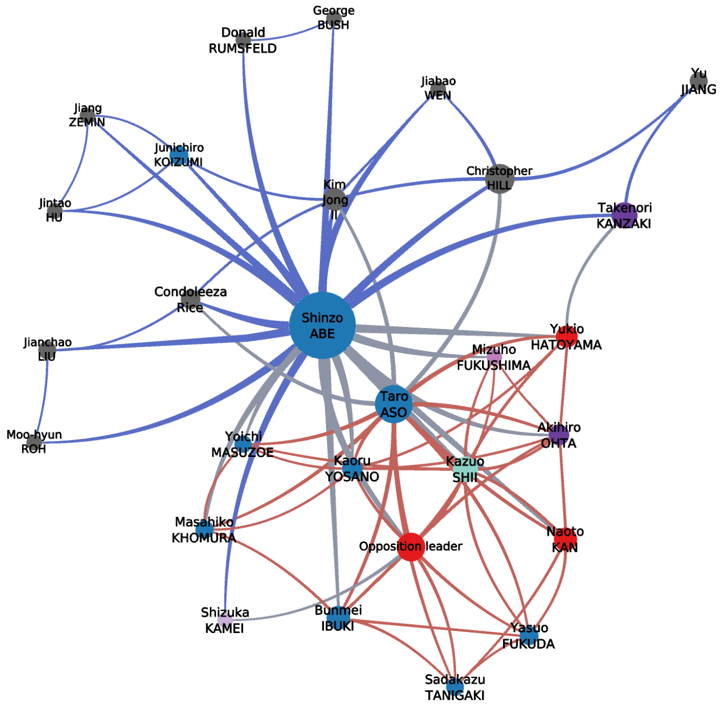

(d) *Abe 1: 2006/09/26 – 2007/09/26*

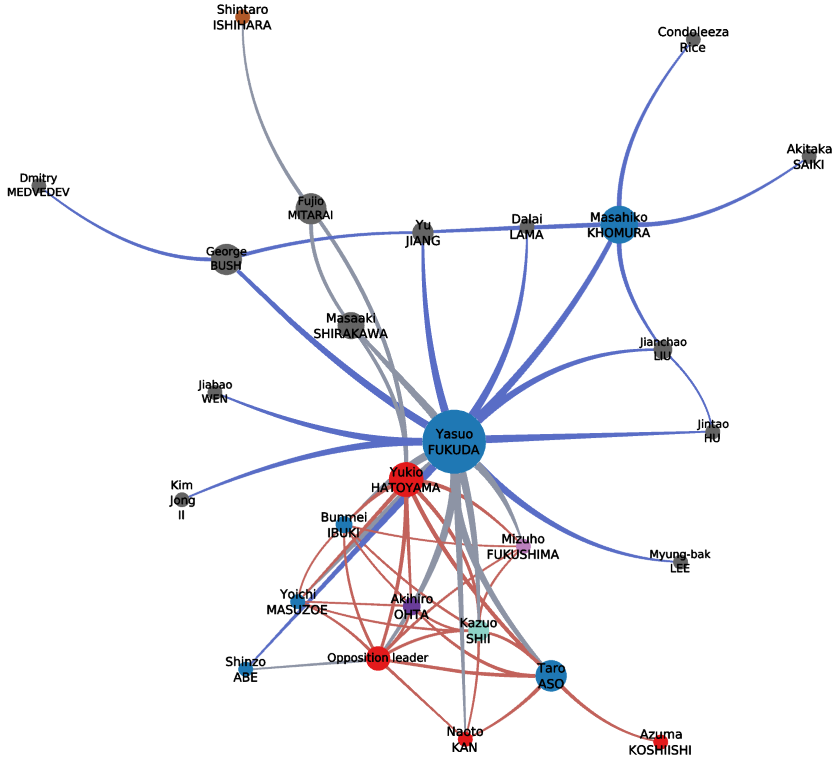

(e) *Fukuda: 2007/09/26 – 2008/09/24*

Figure 13 *The networks are better seen zoomed*. From *Koizumi1* (a) to *Noda* (i), the networks with the Louvain communities highlighted – red and blue links depending on the PM's and OL's affiliation (respectively DPJ and LDP) – purple links if both figure belong to the same community – grey links are out of these communities. The size of a node represents its centrality, nothing encode the size/color of an edge.

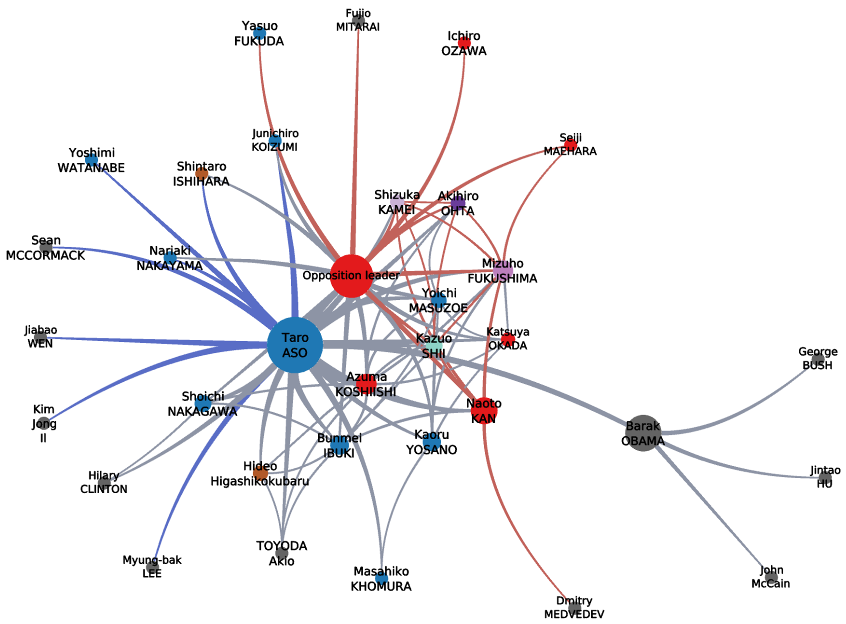

(f) *Aso: 2008/09/24 – 2009/09/16*

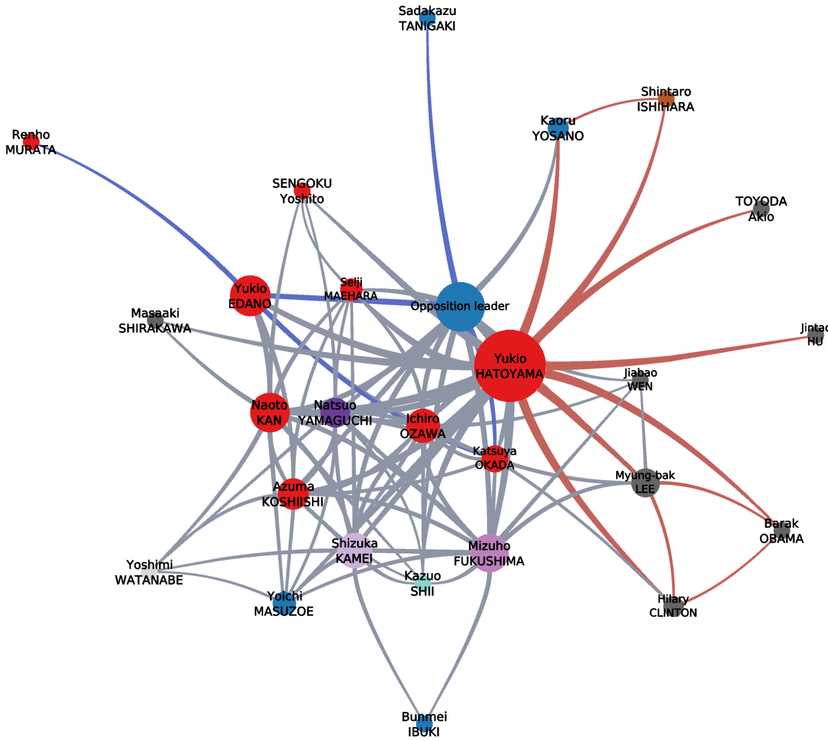

(g) *Hatoyama: 2009/09/16 – 2010/06/08*

Figure 13 *The networks are better seen zoomed*. From *Koizumi1* (a) to *Noda* (i), the networks with the Louvain communities highlighted – red and blue links depending on the PM's and OL's affiliation (respectively DPJ and LDP) – purple links if both figure belong to the same community – grey links are out of these communities. The size of a node represents its centrality, nothing encode the size/color of an edge.

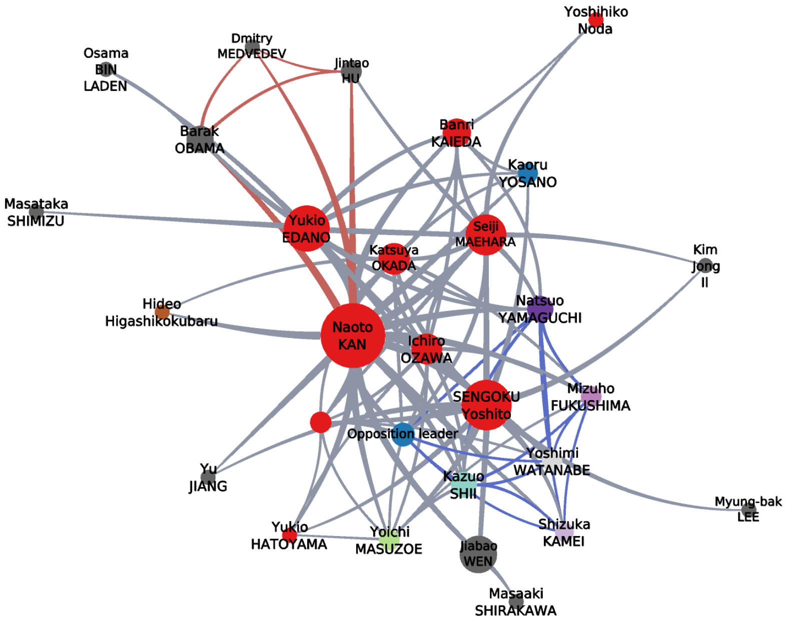

(h) *Kan: 2010/06/08 – 2011/09/02*

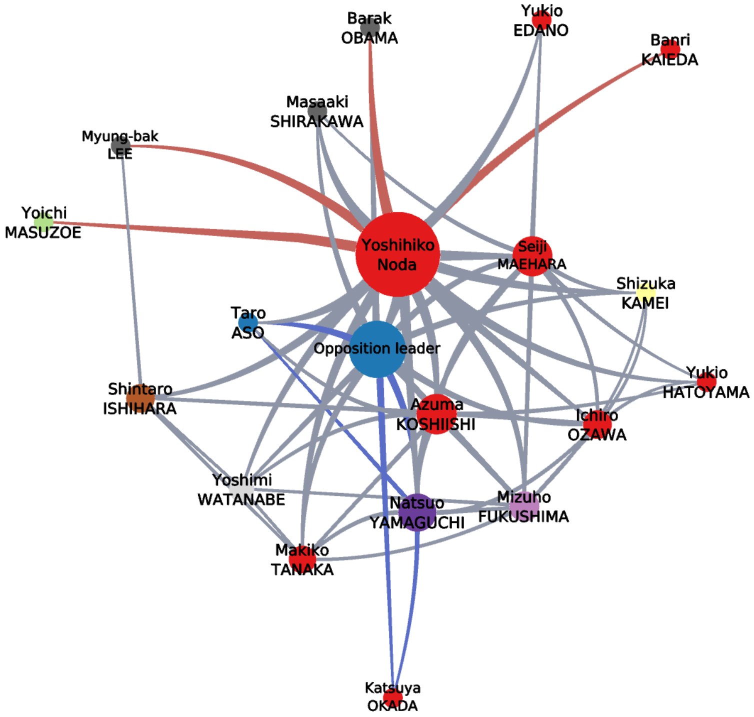

(i) *Noda: 2011/09/02 – 2012/12/26*

Figure 13 *The networks are better seen zoomed.* From *Koizumi1* (a) to *Noda* (i), the networks with the Louvain communities highlighted – red and blue links depending on the PM's and OL's affiliation (respectively DPJ and LDP) – purple links if both figure belong to the same community – grey links are out of these communities. The size of a node represents its centrality, nothing encode the size/color of an edge.
